# Aerosols’ variability and their relationship with climatic parameters over West Africa

**DOI:** 10.1007/s10661-023-11204-x

**Published:** 2023-05-15

**Authors:** Michael Ochei, Ayodeji Oluleye, Ralf Wolke, Dawn Pratt, Teeda Njie

**Affiliations:** 1 Department of Meteorology and Climate Science, Federal University of Technology, Akure, Nigeria; 2 Department of Modeling and Atmospheric Processes, Leibniz Institute for Tropospheric Research, Leipzig, Germany; 3 West African Science Service Center On Climate Change and Adapted Use (WASCAL), DRP-WACS, Akure, Nigeria; 4 Institute of Marine Engineering and Sciences–ISEMAR, Technical University of the Atlantic, São Vicente, Cape Verde; 5 Department of Mathematics, University of The Gambia, Farababanta Campus, The Gambia

**Keywords:** Seasonal variability, Aerosols, Climatic parameters, West Africa

## Abstract

Aerosols’ influences on Earth’s climate have been documented by several authors. This ranges from scattering and reflecting of shortwave radiation (direct effect) which is also regarded as the “Whitehouse Effect,” to the ability to act as condensation nuclei (indirect effect) which results in cloud droplet formation. This broad summary of aerosol’s effect on earth’s climate has in turn affected some other weather variables either positively or negatively depending on people’s perspectives. This work was done in a view to ascertaining some of these claims by determining the statistical significance of some certain aerosol’s relationships with some selected weather variables. This was done over six (6) stations across the West African region to represent the climatic zones from the rainforest around the coasts to the desert of the Sahel. Data used consist of aerosol types (biomass burning, carbonaceous, dust, and PM_2.5_) and climatic types (convective precipitation, wind speed, and water vapor) over a period of 30 years, with the python and ferret programs explicitly used for the graphical analyses. Climatologically, locations close to the point source seem to record more of the presence of the pollutants than the farthest ones. Results indicated that aerosols were more pronounced in the dry months of NDJF over the rainforest region depending on the latitudinal position of the location. The relationship result showed a negative correlation between convective precipitation and aerosols, except carbonaceous. But the strongest relationship can be found between water vapor and the selected aerosol types.

## Introduction

It is a known fact that the resultant aerosol particles from human activities (anthropogenic) such as biomass burning and carbonaceous have contributed substantially to the global mean aerosol burden since the pre-industrial era. Aerosol particles can be said to affect climate systems via different mechanisms. At the top of the list is the direct effect which is a result of solar radiation reflected back to space; absorption of solar radiation by mineral dust to warm the atmospheric aerosol layer, thereby hindering cloud formation or causing cloud droplets to evaporate—semi-direct effect; and the ability to act as condensation nuclei for cloud—indirect effects.

Studies have shown several relationships between different types of aerosols and some weather parameters or events, for example, squall line triggers dust mobilization (Knippertz, 2008, Knippertz et al., 2009, Schepanski et al., 2007, 2009), sub-Saharan aerosols serving as cloud condensation nuclei (CCNs) which reduces cloud droplet effective radius, suppresses drizzle formation, and changes cloud lifetime—highly referred to as aerosol indirect effects (AIEs) (Twomey, 1977; Seinfield & Pandis, 2006), among other relationships. But none has shown the relationships at certain statistical significance levels of certain aerosols (regarded as major ones over West Africa) to some weather parameters. The correlation and statistical significance of some aerosols (biomass burning, carbonaceous, dust, and PM_2.5_) against each of these weather variables (wind speed, convective precipitation, and amount of water vapor in the atmosphere) were considered in this research, as well as the spatial distribution of those aerosol types over West Africa. This choice of these weather parameters was a result of their importance to convective system propagation, precipitation deposition, and precipitable cloud formation.

Of meteorological interest in the atmosphere are particles as small as molecules and of up in size through clouds and precipitation elements to giant hailstones. But over the last few years, the understanding of climate has hinged on the recognition of atmospheric aerosol. Most, if not all, airborne particles may exert a net cooling on the earth’s surface bar greenhouse gas CO_2_ which contributes to global warming. The introduction of the “Whitehouse Effect” was done to summarize some atmospheric radiation effects that are driven by atmospheric aerosol (Schwartz, 1996). The presence of aerosols in the atmosphere can impact radiation (e.g., reflection and scattering), which can lead to changes in other variables (e.g., air temperature and relative humidity), and this in turn can lead to adverse effects on human health and the environment. To have a better knowledge of this, identification of the presence of aerosols on a radiative budget (Wong et al., 2012) can quantify both the direct and indirect effect of aerosols and climatic variables (Wang et al., 2014; Xing et al., 2017).

In reference to precipitation, aerosols affect cloud hydrometers by acting as cloud condensation nuclei (CCN) or ice-nucleating particles (INP). While the CCN can lead to new droplet formation and also increase the number of cloud droplets and cloud albedo (Twomey, 1974), the INP on the other hand, a rare type of aerosol, can trigger droplet freezing. After droplets freeze, the resulting ice particles tend to grow at the expense of existing cloud droplets, eventually leading to precipitation (Bergeron, 1935; Findeisen et al., 2015; Wegener, 1911). On the other hand, the ability of precipitation to be suppressed by pollution aerosols has been investigated in recent years (Huang et al., 2007; Giorgi et al., 2003).

Clouds are the key element for the understanding of the global radiation budget because of the role it plays in the amount of shortwave radiation to the Earth’s surface or atmosphere. The formation of clouds can be linked to the process that involves cloud condensation nuclei—which arise from anthropogenic emissions caused by man. Early research from Haywood et al. (2001) over Cape Verde Islands revealed an aerosol radiative forcing of −60 to −35 Wm^−2^ for mineral dust. Aerosols and clouds still pose the largest uncertainty to estimates and interpretations of Earth’s changing energy budget despite the substantial effort taken in the last decades to improve the knowledge about the role of aerosols in the climate systems (IPCC, 2013). Mineral dust particles, among others, are very important due to their contribution to about half of the global annual particle emissions by mass (Hinds, 1999; Huneeus et al., 2011); with a significant impact on the radiation budget of the Earth by scattering, absorption, and emission of solar and terrestrial radiation (Sokolik et al., 2001; Tegen, 2003; Balkanski et al., 2007). Saharan dust is also assumed to influence the formation of tropical cyclones in the mid-Atlantic (Evan et al., 2006; Wu, 2007; Sun et al., 2008).

Going by the aforementioned reviews which have shown several impacts of aerosols on weather variables either in a positive or negative direction, it is pertinent to show how some types of aerosols relate with some selected weather variables in different ways. Hence, this work was done to establish the spatial variability of four (4) selected aerosol types (biomass burning, carbonaceous, dust, and PM_2.5_) and also determine the relationship that exists between the aerosol types and some weather variables (amount of water vapor in the atmosphere, wind speed, and convective precipitation).

## Study area

**Fig. 1:**
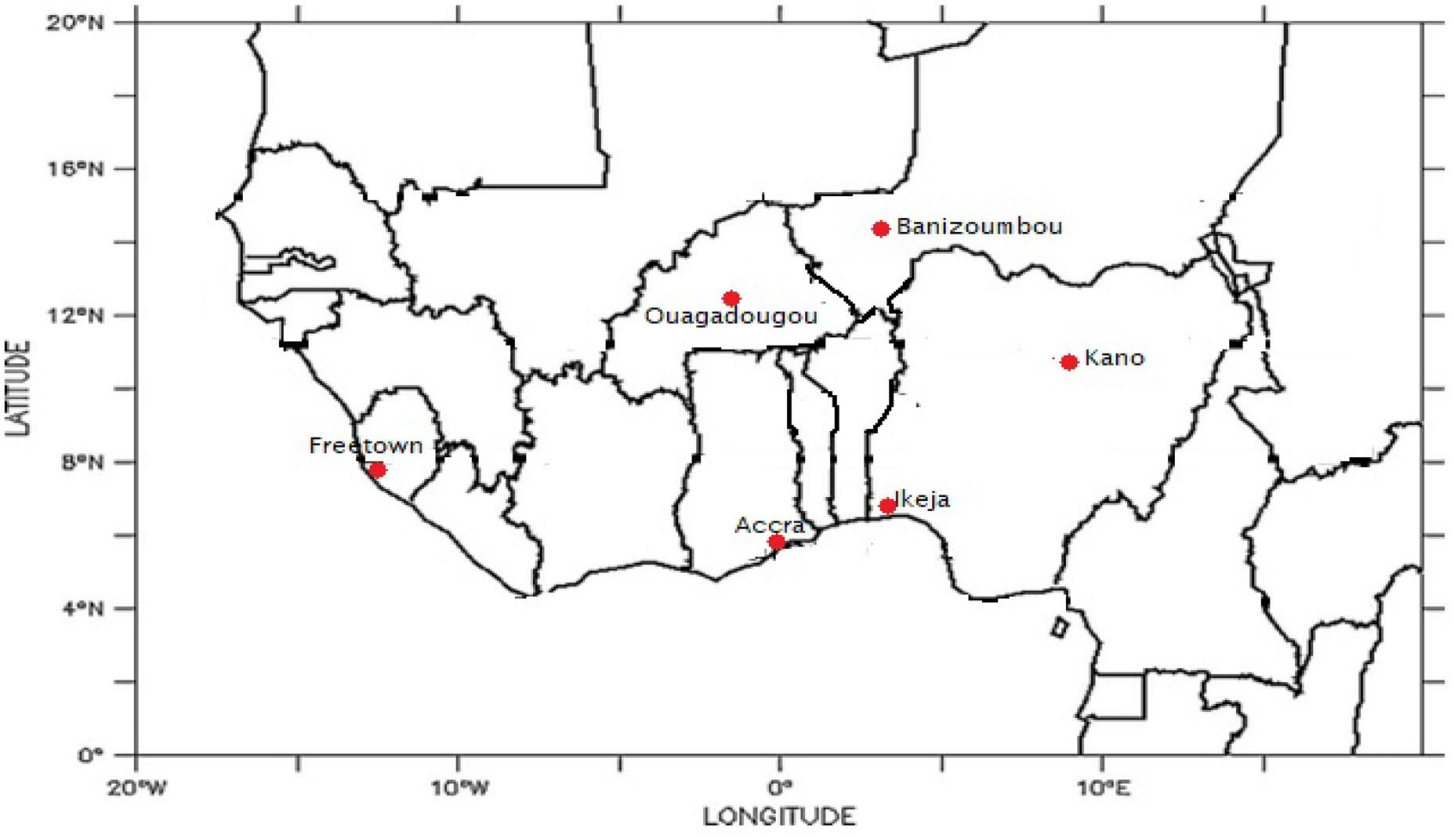
Map of West Africa showing the study areas (red circle)

West Africa lies roughly between latitudes 2° N–25° N and longitudes 25° W–25° E, with an estimated area cover of 6,140,000 km^2^ (Fig. 1). The region consists of different climatic regions, which range from the Sahel through the Savannah to the Rainforest. Bounded to the south is the Gulf of Guinea, to the north are about three (3) countries combined, namely, Mauritania, Mali, and Niger; to the eastern limits is the Mount Cameroon/Adamawa Highlands; and the Atlantic Ocean to the western boundary. The region is predominantly under the influence of two (2) air masses all year round, namely, northeast trades which bring about the cold Harmattan dry season and the moist-laden southwest monsoon which brings about the wet season (Omotosho, 1985). These winds determine the season of the year and are influenced by the position of the Inter-Tropical Discontinuity (ITD). South and north of the ITD are laden with moisture and dust respectively at any time of the year (Ochei et al., 2015). Dust transport towards the coast of the Atlantic Ocean by the wind is dependent on the quantity raised at the source point.

## Data and methodology

In the consideration of aerosol prevalence over the study area, the time of the year is very important, just as the wind direction and strength are vital. It is a well-known fact that dust occurrence is prevalent during the dry season when the ITD would have retreated to its southernmost position close to the Atlantic Ocean, thus giving way to the presence of northeast trades all over the continent. Though some episodes have been recorded during the rainy seasons, they are seen as an anomaly because of certain deviations from the norms of that particular period. But in considering the seasonal and inter-seasonal variability (in its spatial form) of some selected aerosol types, 30 years (1988–2017) monthly reanalysis datasets from Giovanni for aerosol (biomass burning, carbonaceous, dust, and PM_2.5_) were extracted for six locations, namely, Accra, Banizoumbou, Freetown, Ikeja, Kano, and Ouagadougou. These datasets were extracted at a resolution of 0.5° × 0.625°. Python was used to carry out all analyses. The seasonal spatial graphs were done in order to determine the distribution of each of the selected aerosol types on a quarterly basis, by dividing the months of the year into DJF, MAM, JJA, and SON. This is done in order to have knowledge of the seasonal distribution of the aerosol types. The correlation graphs were used to determine the relationships between each climatic variable considered and the selected aerosol types, and the probability value, at a specific significant level, was used to either reject the null hypothesis in favor of the alternate, thus making the relationship between two variables statistically significant or failed to reject the hypothesis, thus making the correlation not statistically significant. The correlation and *p* value were calculated using

1 $$r=\frac{\sum (xi - x)(yi -y)}{\sqrt{\sum \left(xi - x\right)2\left(yi -y\right)2}}$$

2 $$t=\frac{r x \sqrt{n -2}}{\sqrt{1 -r2}}$$

where *r* is the correlation; *x* is the independent variable; *y* is the dependent variable; and *n* − 2 is the degree of freedom.

### Seasonal and inter-seasonal spatial distribution of aerosols over West Africa

The availability of biomass in the seasonal months of December/January/February around the stretches of the Atlantic bounded areas can be said to be a result of two possibilities. The first result can be deduced to be the pre-planting activities in expectation of the onset of the rainy season in the region. Another possible scenario can be adduced to the role of the prevailing easterly wind which can aid the transportation of the pollutant from Central Africa to Western Africa. The large presence of biomass burning in central Africa can be attributed to the fact that the area is said to have an estimated carbon emission from deforestation in 1980 to range from 17.9 to 22.5million tonnes, accounting for about 20% of the total carbon emissions over the entire area of tropical Africa (Brown et al., 1989; Hall & Uhlig, 1991). It should be noted that the prevailing wind during this period of the year is easterlies. Hence, the second theory or possibility can be said to be truer in this scenario. The months of March/April/May showed the activity of biomass spotted around Freetown. Freetown may be a coastal zone but partly share the characteristic of the Savannah region in which the onset of rain has been pegged on average to be around the month of May. Hence, the presence of biomass can be adduced to pre-planting season activities as the onset of rainfall over this location is not the same as in other rainforest zones. In the consideration of June/July/August, there was no evidence of biomass activity over the study area. But patches were discovered in the transition months of September/October/November depending on the latitudinal position.

Carbonaceous presence in the atmosphere is well pronounced in the Harmattan months of DJF, spanning the whole length of the coastal cities. This is in a similar pattern to biomass burning and thus supports the study of van der Werf et al. (2010) which estimated around 2PgC/year of global CO_2_ emissions due to about 4% of the global land area subjected to burning (Giglio et al., 2013). Though studies may have shown similarities in biomass and carbon but comparing the results of both pollutants in the months of March/April/May (MAM), it can be observed that carbon seems pronounced around Ikeja which is not available in biomass burning during the said period. Also, Freetown seems to record an expanded area under carbon prevalence when compared to biomass. The reason for the high concentration of the pollutant at Ikeja and Freetown only within the coastal areas during this period can be attributed to anthropogenic effects. The wet months of June/July/August still showed Ikeja having a concentration of pollutants while Freetown is clear. The continuous presence of carbonaceous in Ikeja all through the year can be attributed to the fact that it is the commercial hub of West Africa with engrossed human and vehicular activities.

**Fig. 2:**
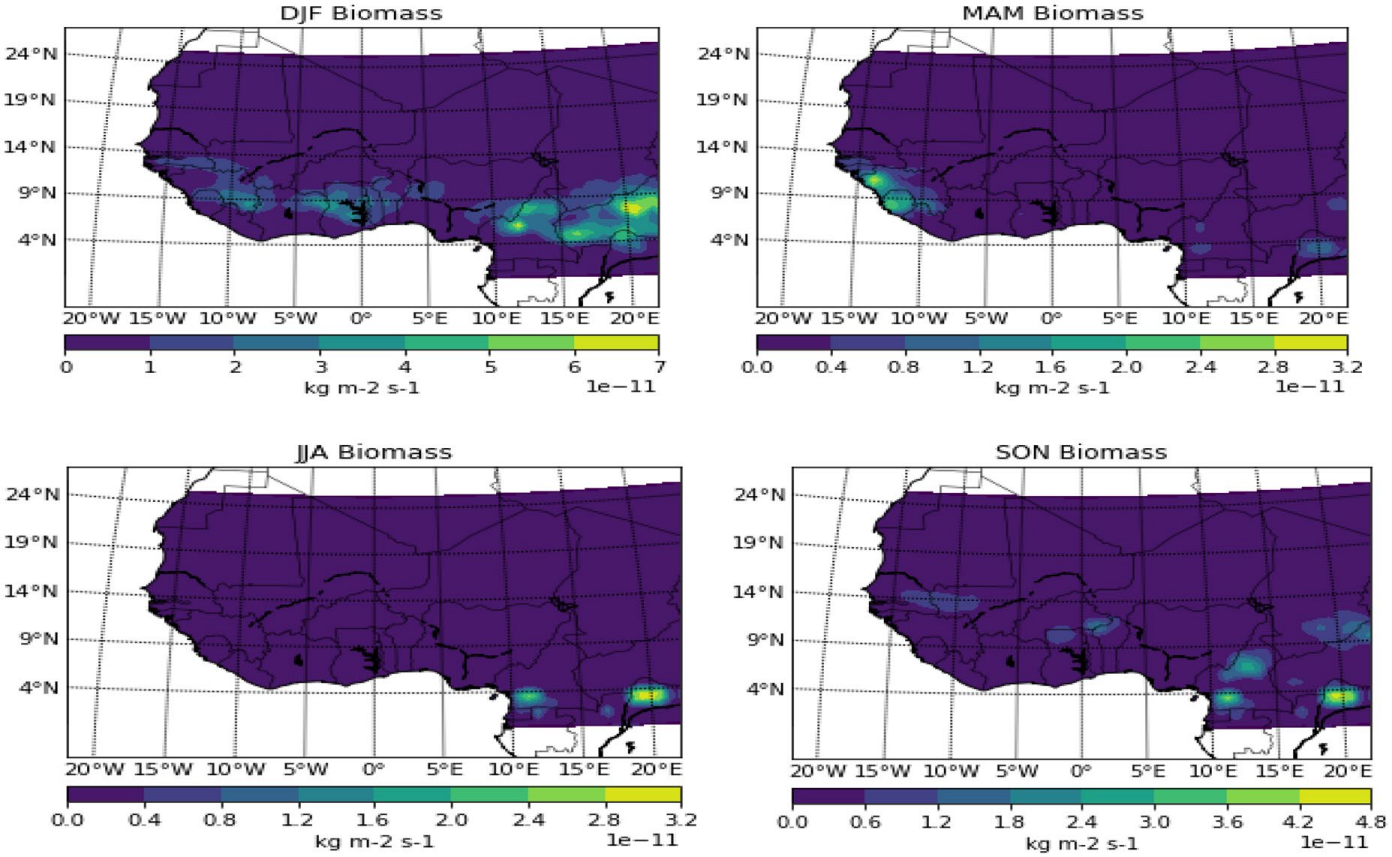
Seasonal variation of biomass burning over West Africa

**Fig. 3:**
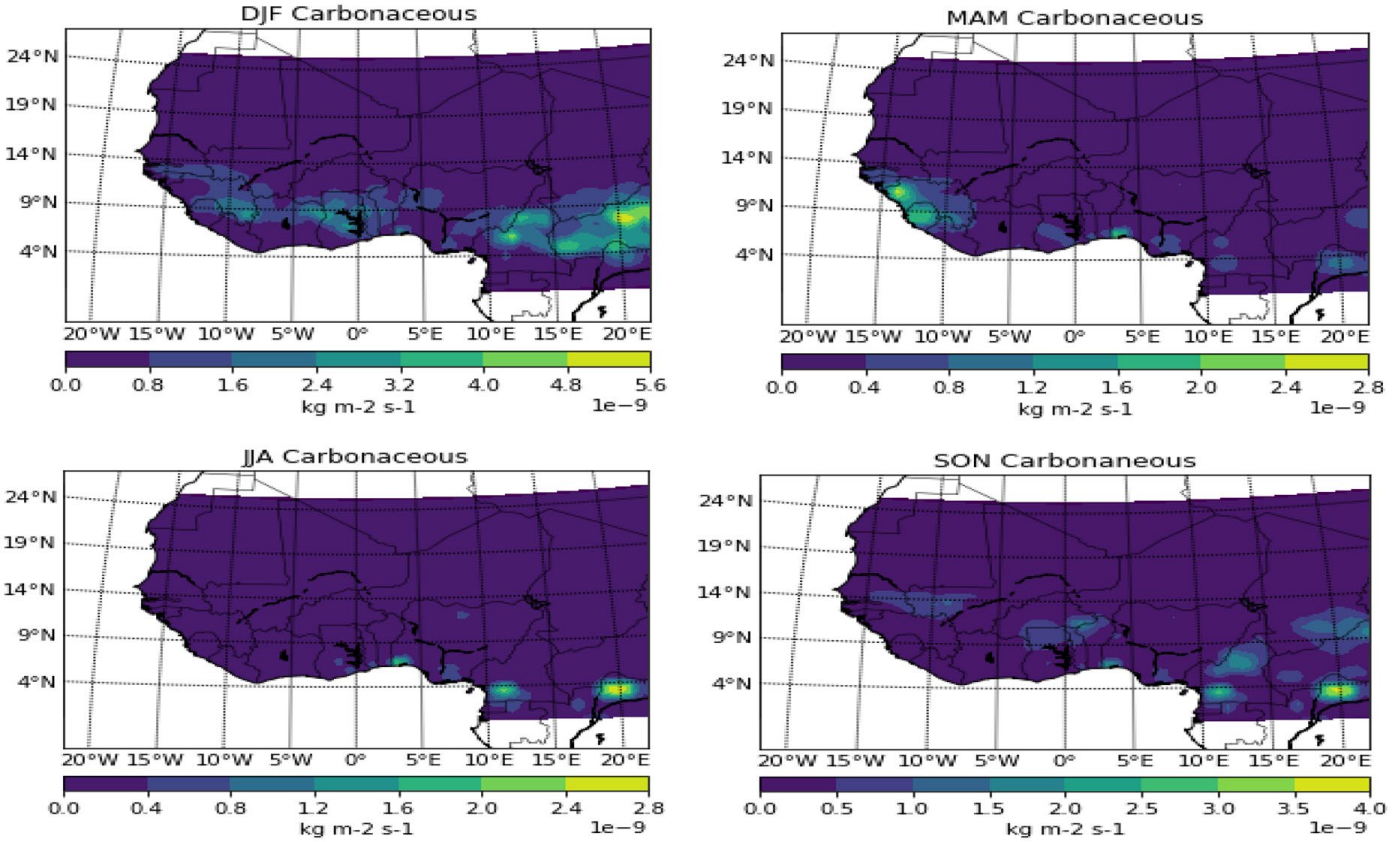
Seasonal variation of carbonaceous over West Africa

**Fig. 4:**
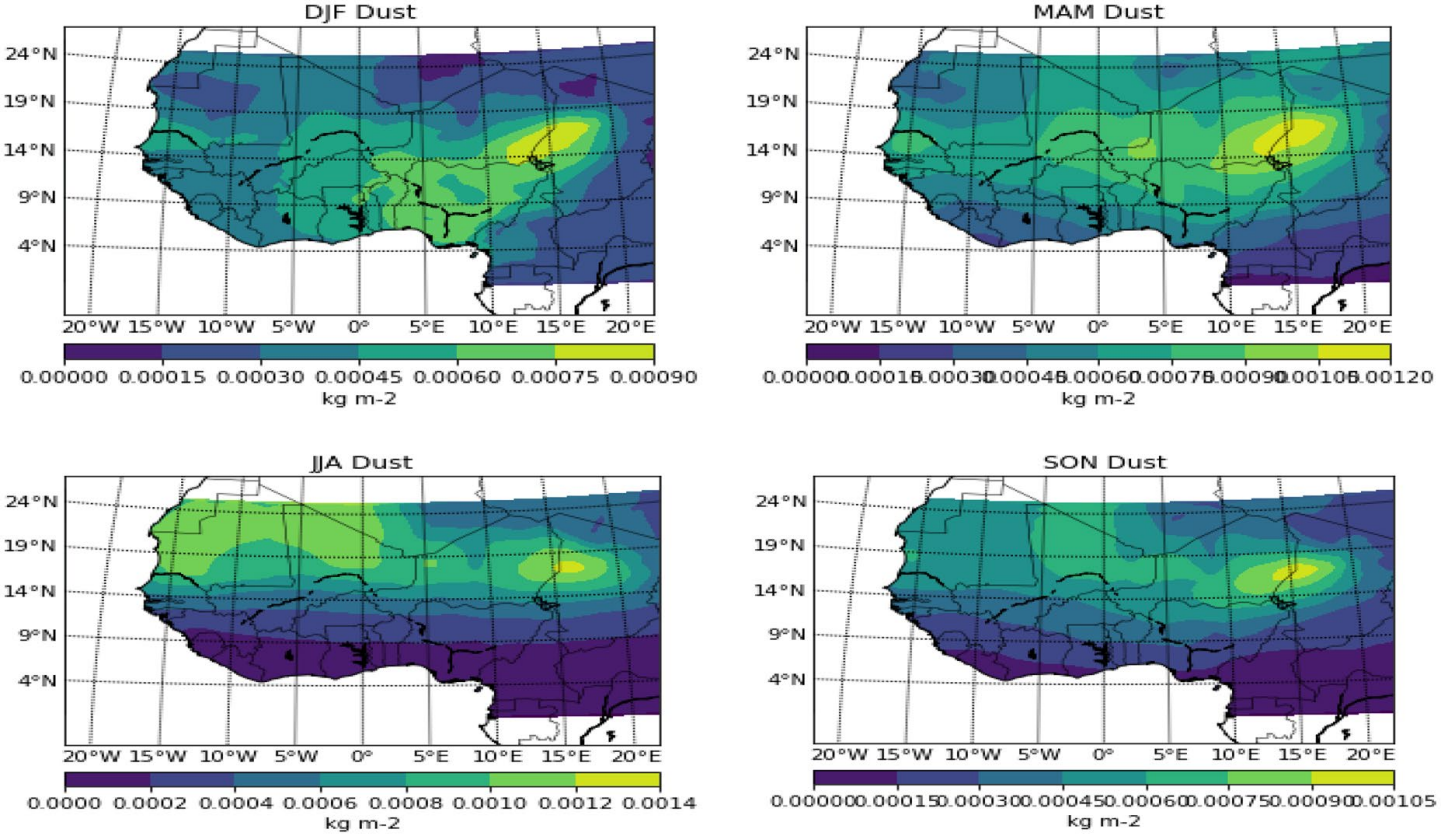
Seasonal variation of dust over West Africa

**Fig. 5:**
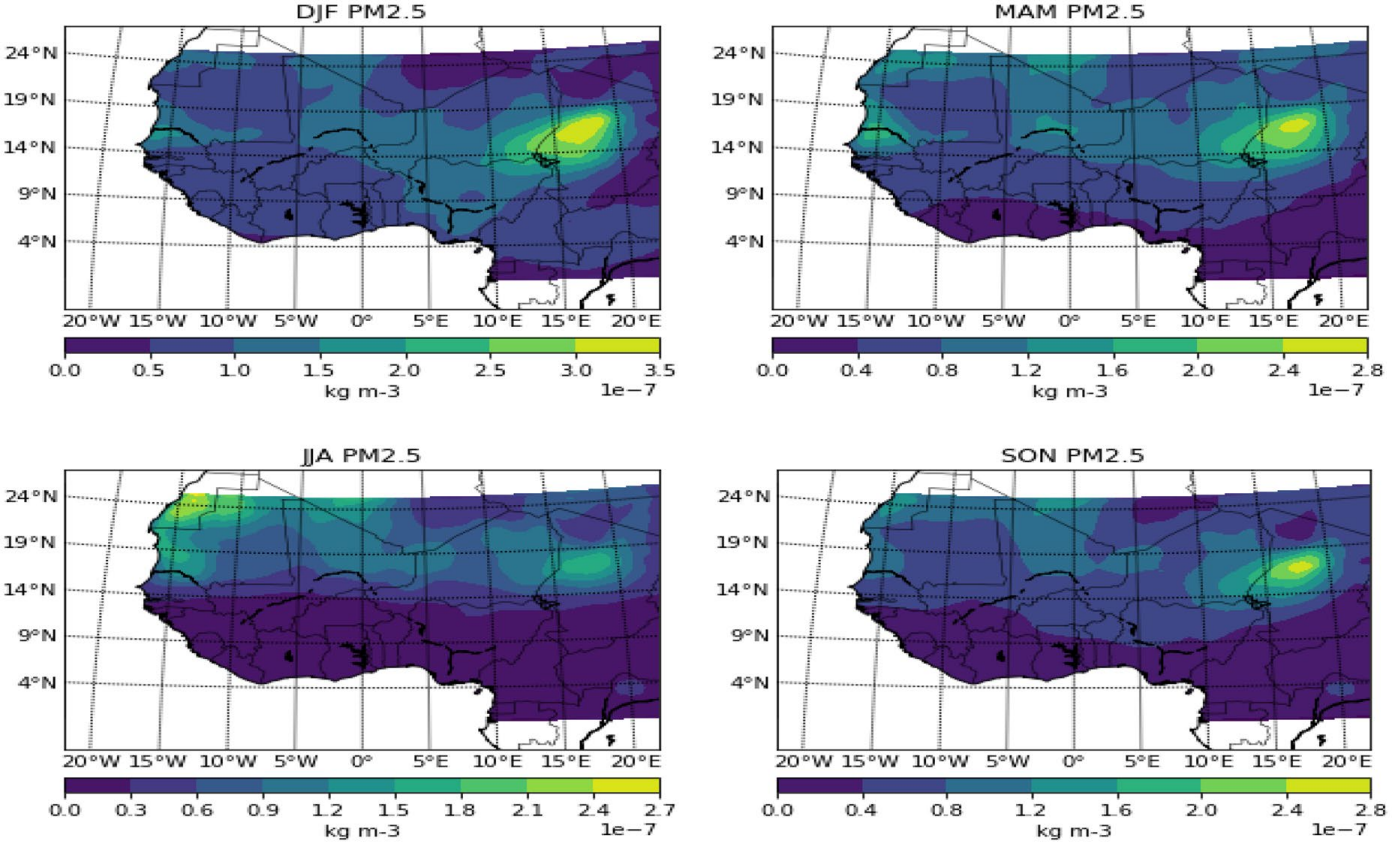
Seasonal variation of PM_2.5_ over West Africa

The Harmattan months of DJF saw the dust spread to the southernmost part of the study area, covering the whole of Nigeria and spreading up to 5° W, and also a significant patch of it around Dakar at 15° N (Figs. 2, 3, 4, and 5). Due to the depth of this pollutant southward, it is often regarded as a thick dust haze (TDH) and reduces visibility (Ochei & Adenola, 2018). The source point showed a well-laden dust event, with a significant amount spreading across Sudan and some parts of the Guinea savannah. The potential “early” onset of the rainforest part in the months of MAM seems to have pushed back the dust further north, and this can be attributed to the northward migration of the ITD, thereby making south of the ITD to be under the influence of moist southwest monsoon (Oluleye & Okogbue, 2013). The month of JJA saw the whole rainforest and Guinea savannah under the influence of convective activities due to the northernmost position of ITD, though some areas north of 15° N (Sahel) and most especially the point source are mostly under the influence of dust activities. The dust seems to be intensifying again and thereby pushing further down the south towards the Atlantic Ocean in the transition month of SON.

PM_2.5_ showed similar features in Harmattan months of DJF and onset months of MAM. Though the coastal axes did not have a record of the pollutant in the onset months, further inland to the north showed a similar pattern with DJF over the Sahel and desert areas. For the wet months of JJA, the pollutant is completely negligible from the equator up to 15° N, but some traces can be seen over the Sahel. The cessation months of SON witnessed the spread of the pollutant towards the southern part, though areas south of 9° N–10° N are not under the influence of the pollutant. The point source of dust in Bodélé depression exhibited a similar pattern with PM_2.5_ and this is in support of De Longueville et al.’s (2010) study which showed that Saharan dust contributes up to 1100 Tg of particulate matter to the annual, thereby making it the most active among all source regions worldwide.

### Correlation between aerosol types and climatic parameters

Several studies have been done to unravel the relationships, vis-à-vis, the effect of aerosol on meteorological variables or the other way around (Figs. 6, 7, 8, 9, 10, 11, 12, 13, 14, 15, 16, 17, 18, 19, 20, 21, 22, and 23). One such study showed that squall lines and density currents which are associated with moist convection are meteorological effects that cause strong winds necessary to trigger dust mobilization (Knippertz, 2008; Knippertz et al., 2009; Schepanski et al., 2007, 2009).

**Fig. 6:**
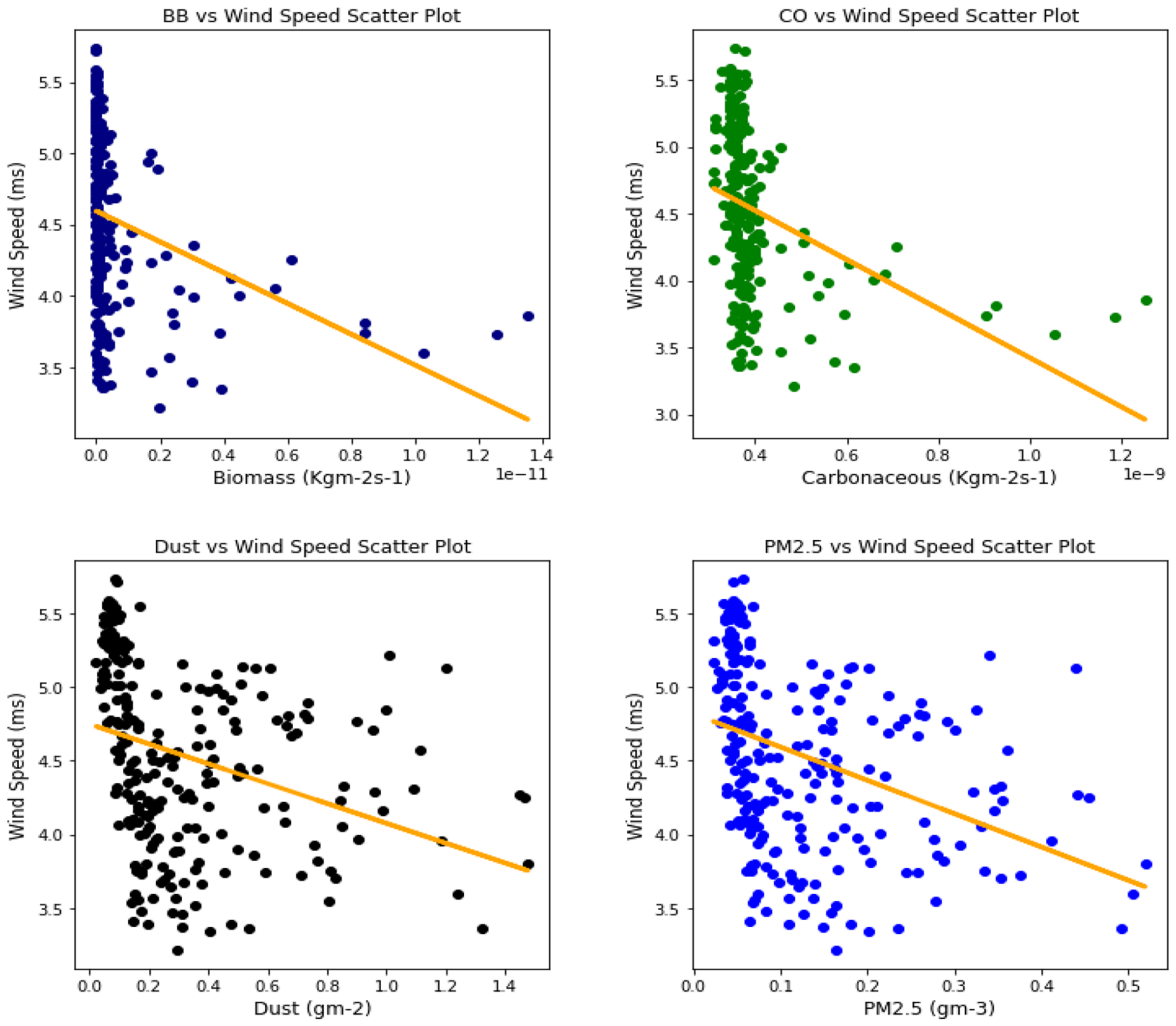
Correl. of aerosols and wind speed over Accra

**Fig. 7:**
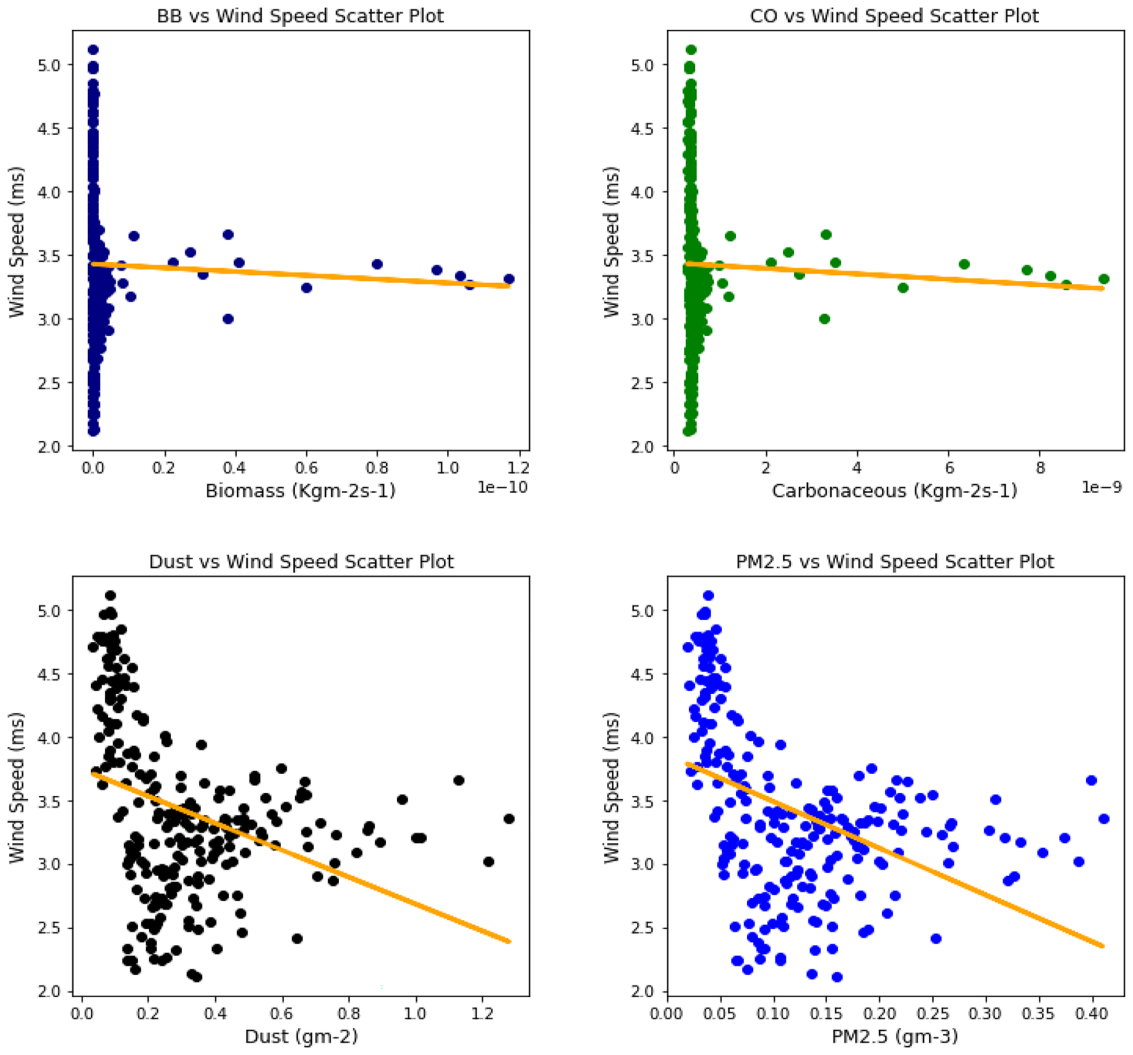
Correl. of aerosols and wind speed over Freetown

**Fig. 8:**
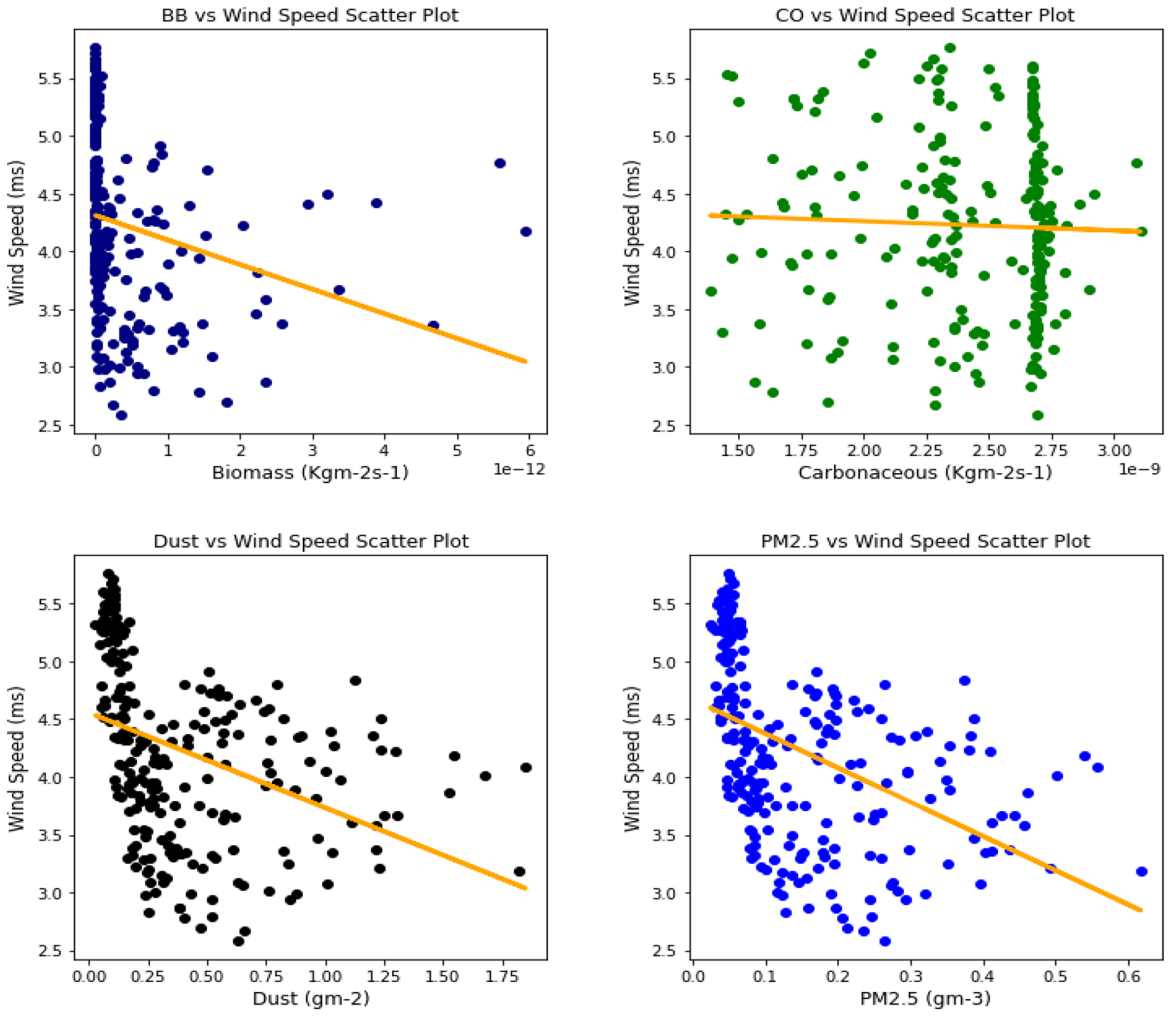
Correl. of aerosols and wind speed over Ikeja

**Fig. 9:**
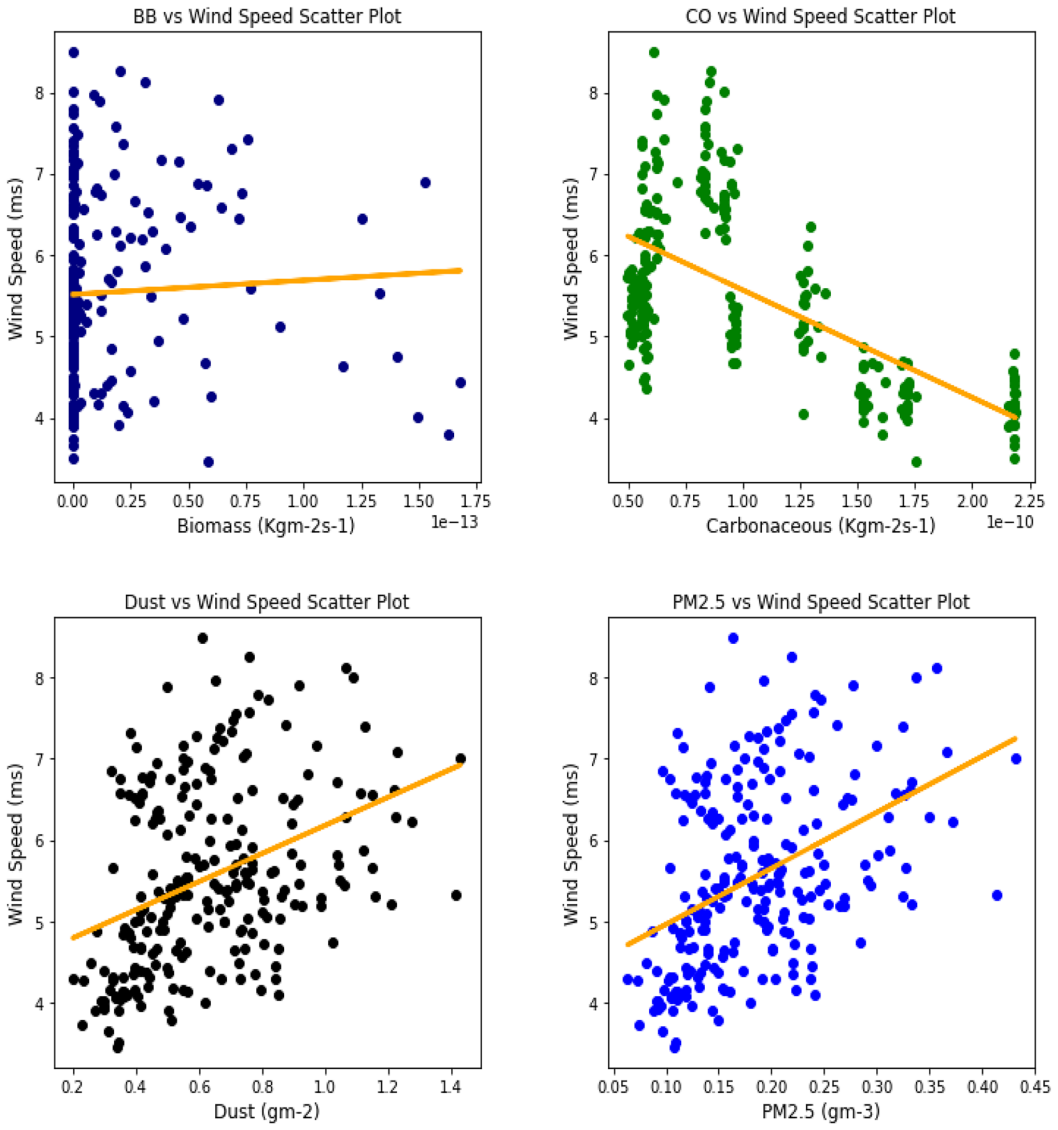
Correl. of aerosols and wind speed over Banizoumbou

**Fig. 10:**
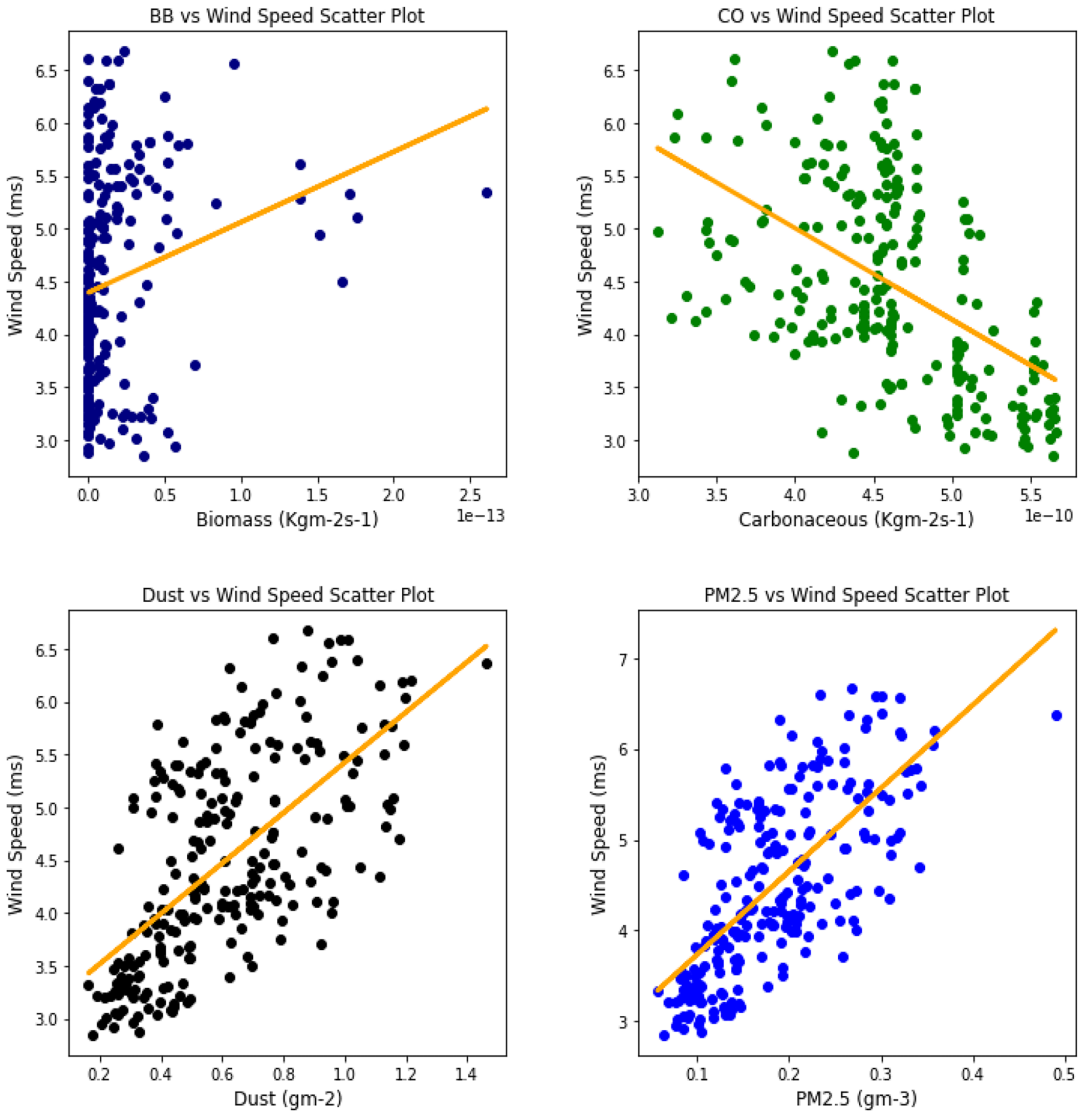
Correl. of aerosols and wind speed over Kano

**Fig. 11:**
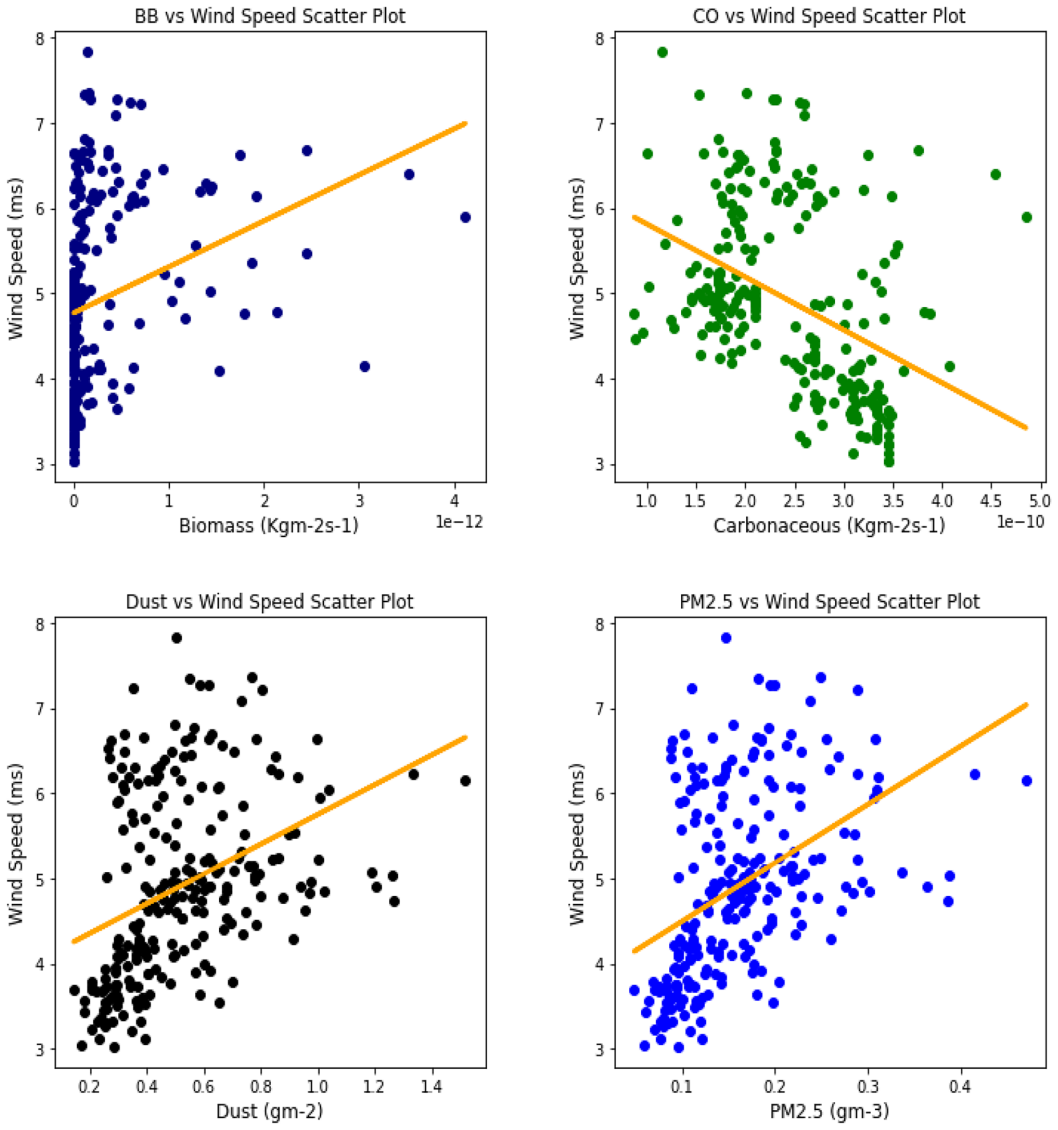
Correl. of aerosols and wind speed over Ouagadougou

**Fig. 12:**
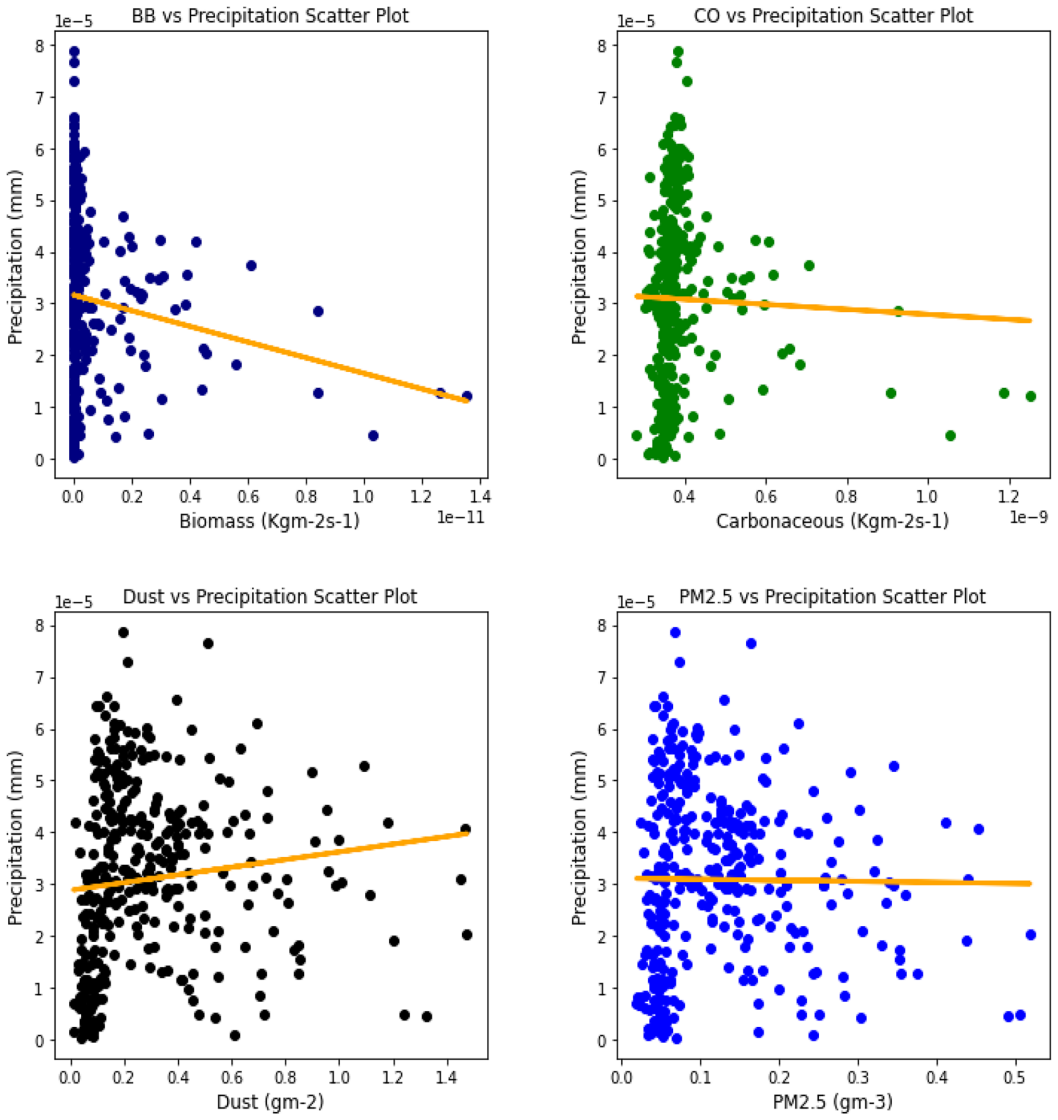
Correl. of aerosols and conv. ppt. over Accra

**Fig. 13:**
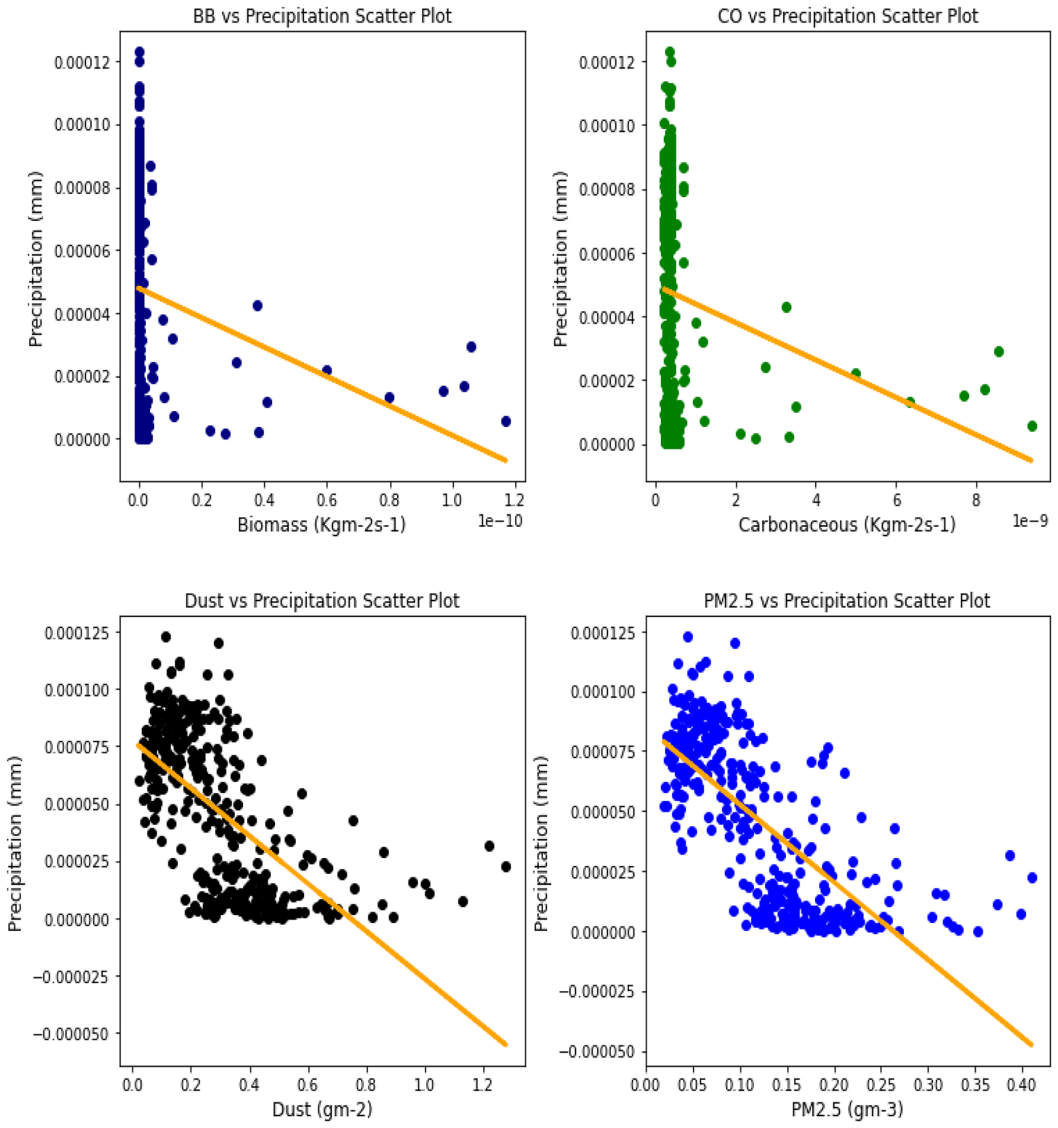
Correl. of aerosols and conv. ppt. over Freetown

**Fig. 14:**
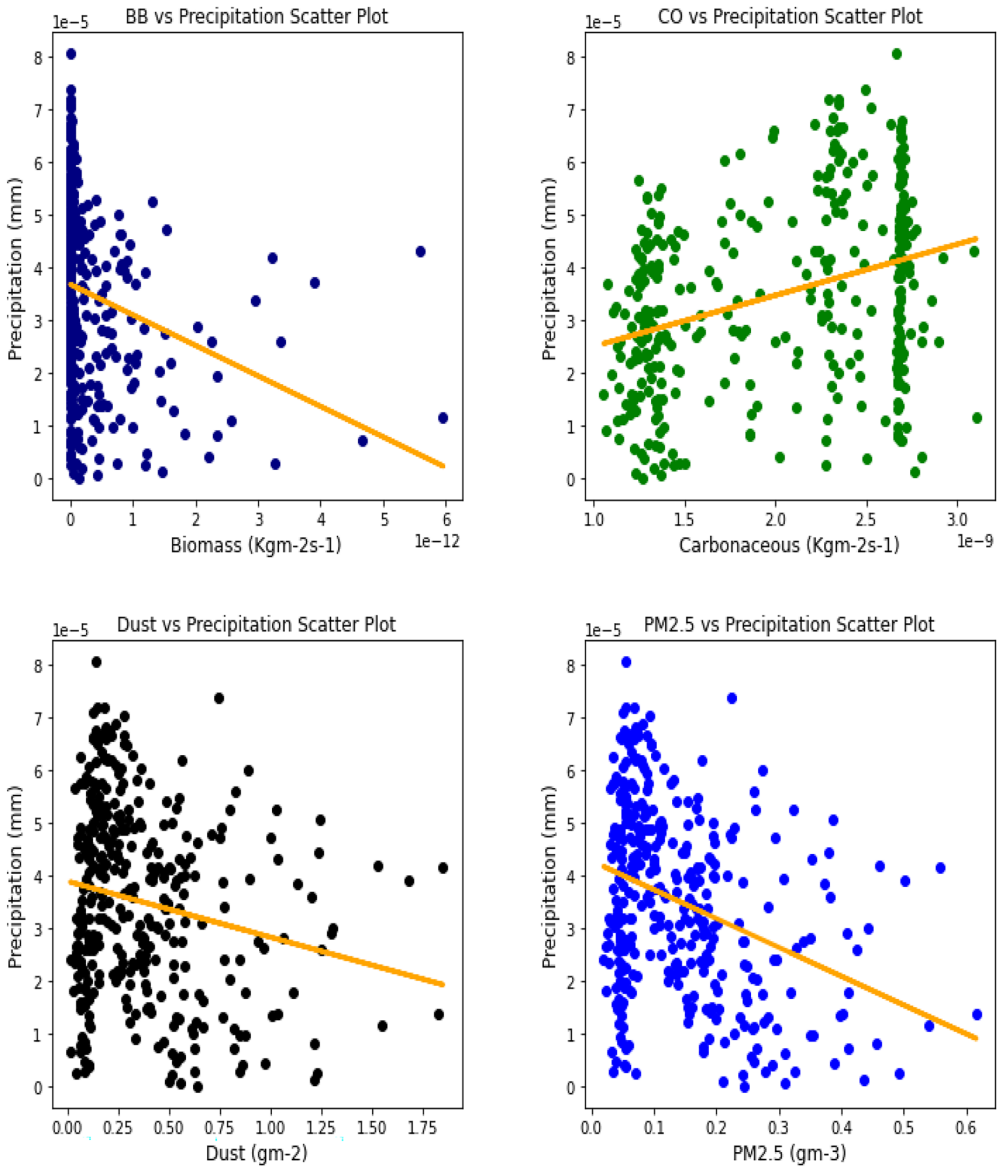
Correl. of aerosols and conv. ppt. over Ikeja

**Fig. 15:**
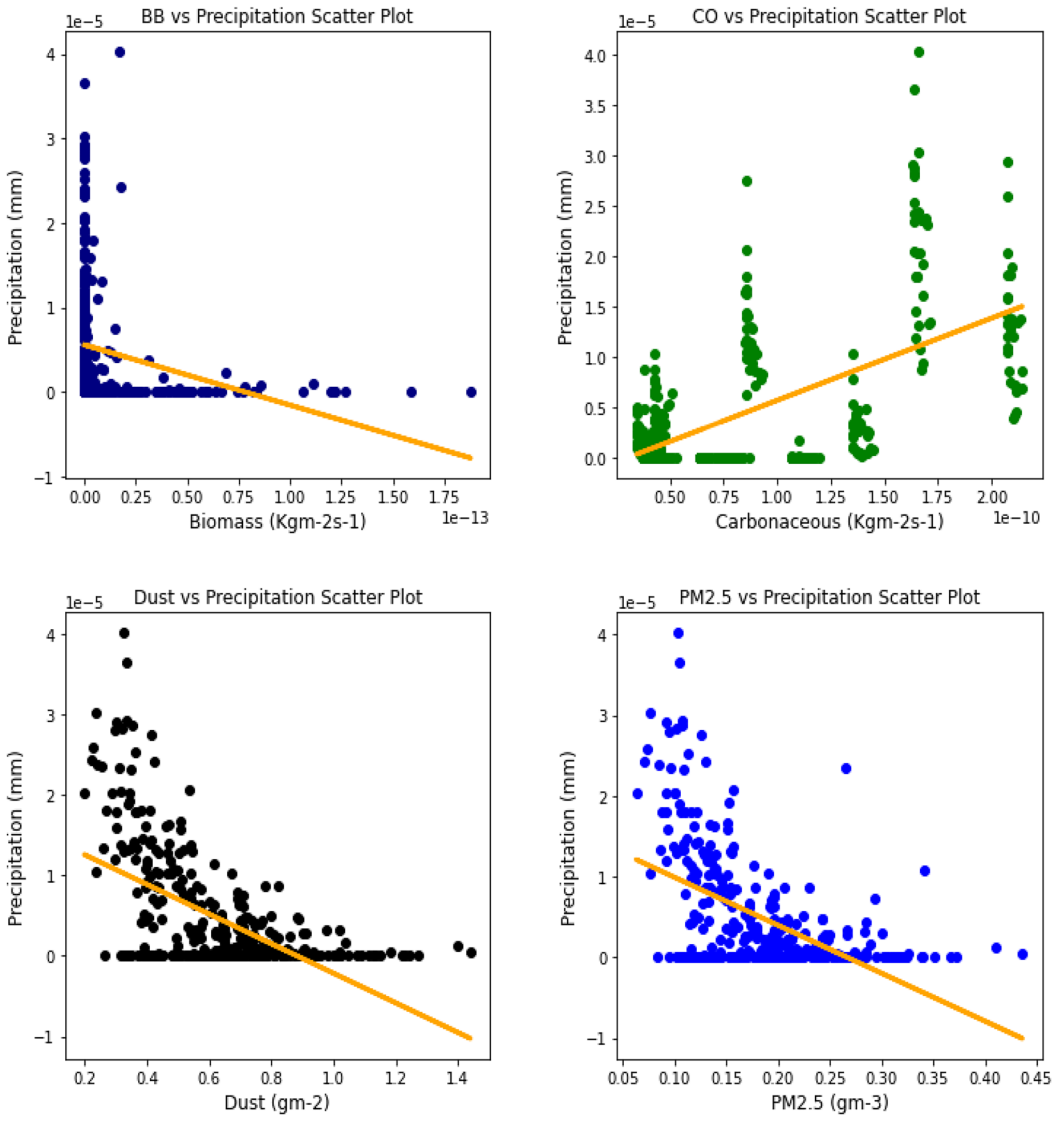
Correl. of aerosols and conv. ppt. over Banizoumbou

**Fig. 16:**
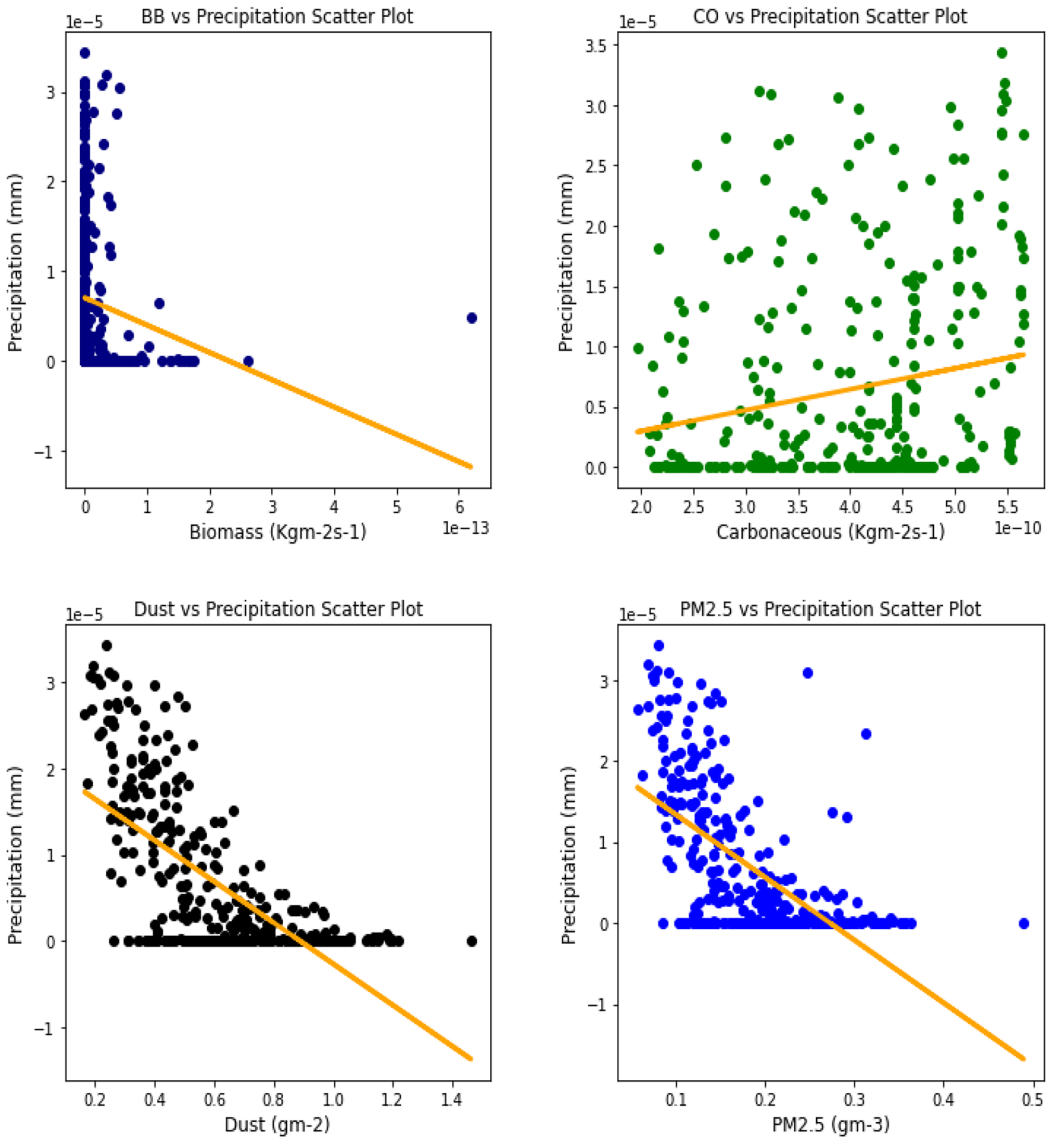
Correl. of aerosols and conv. ppt. over Kano

**Fig. 17:**
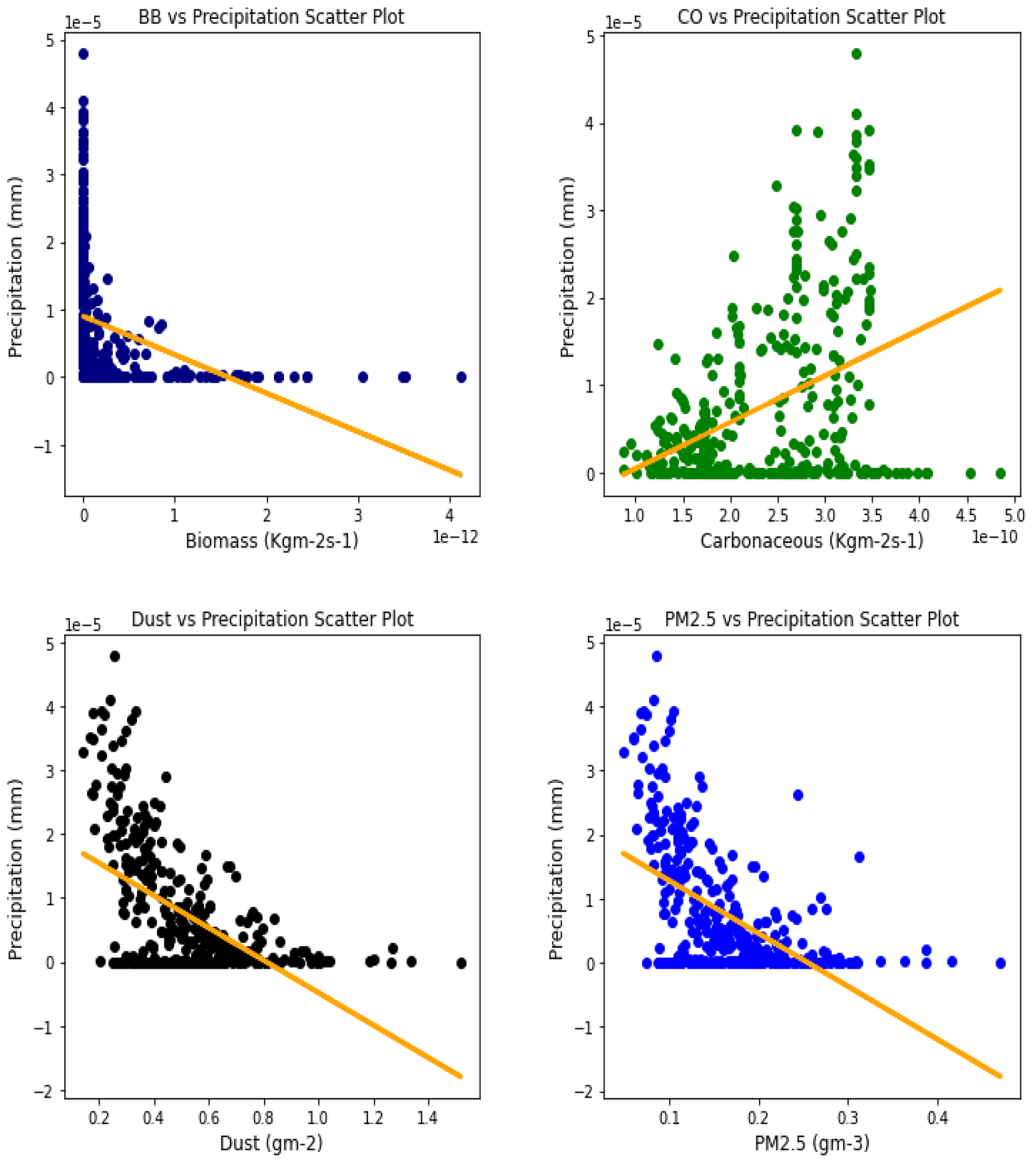
Correl. of aerosols and conv. ppt. over Ouagadougou

**Fig. 18:**
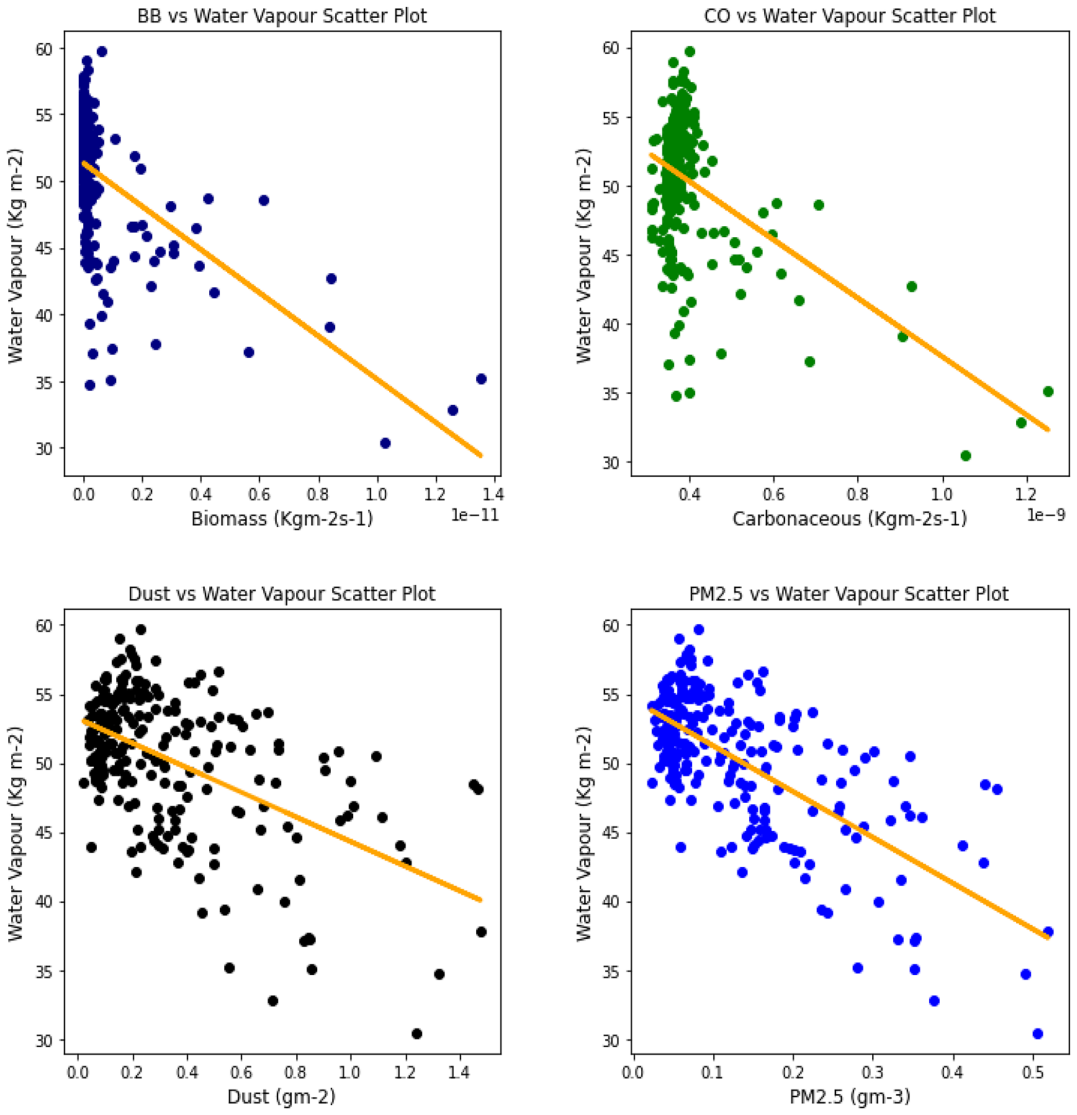
Correl. of aerosols and H_2_O vapor over Accra

**Fig. 19:**
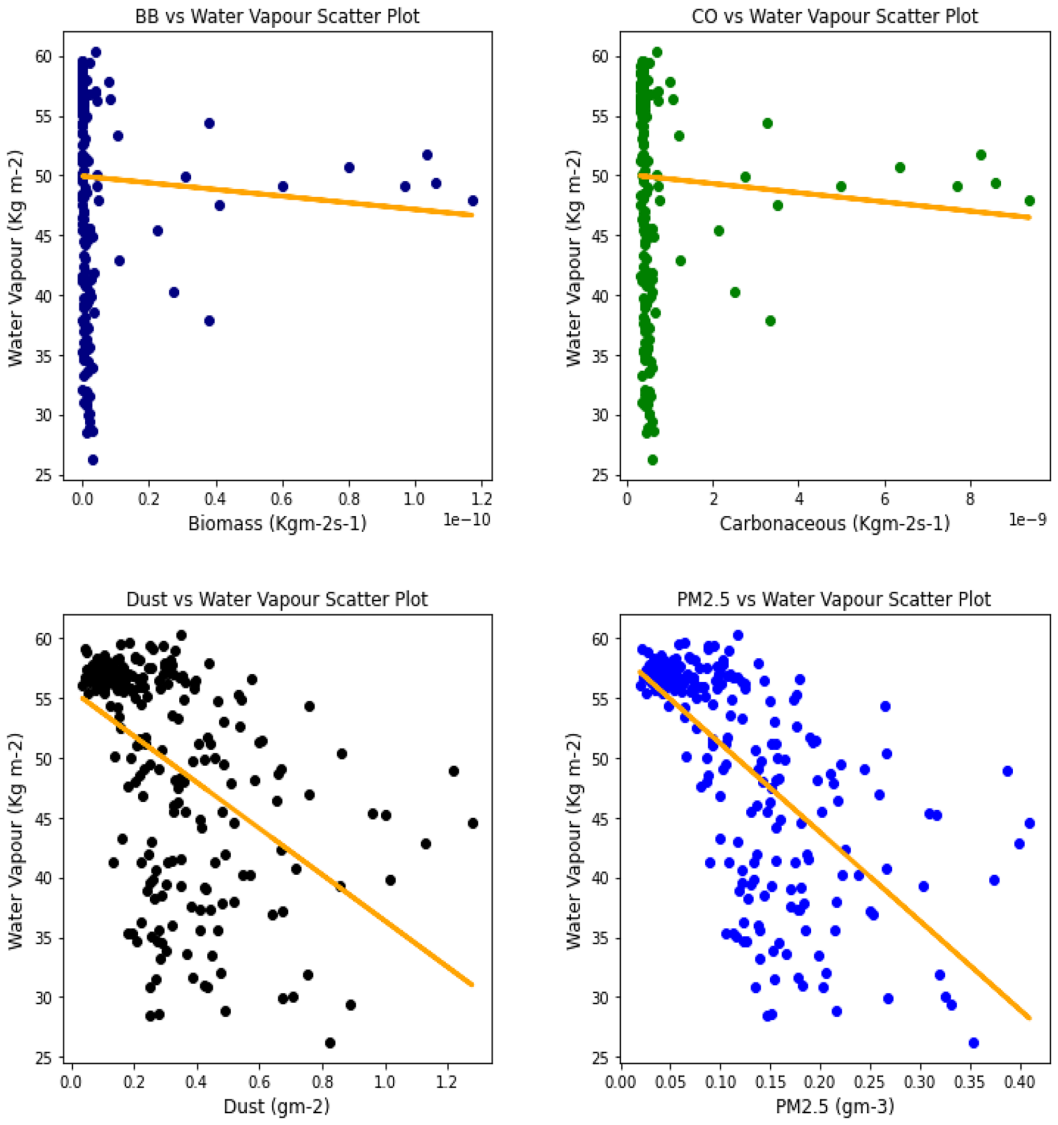
Correl. of aerosols and H_2_O vapor over Freetown

**Fig. 20:**
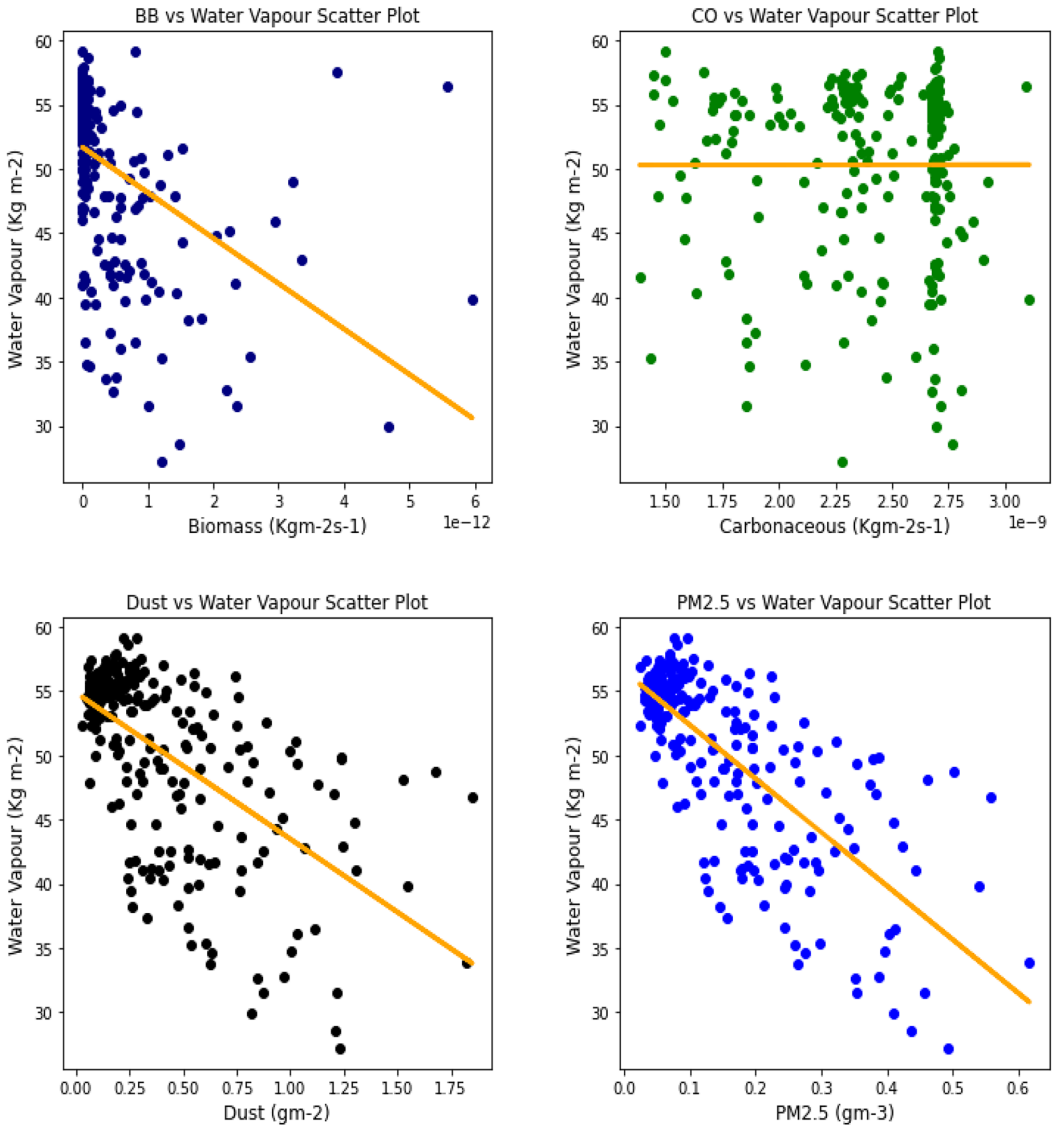
Correl. of aerosols and H_2_O vapor over Ikeja

**Fig. 21:**
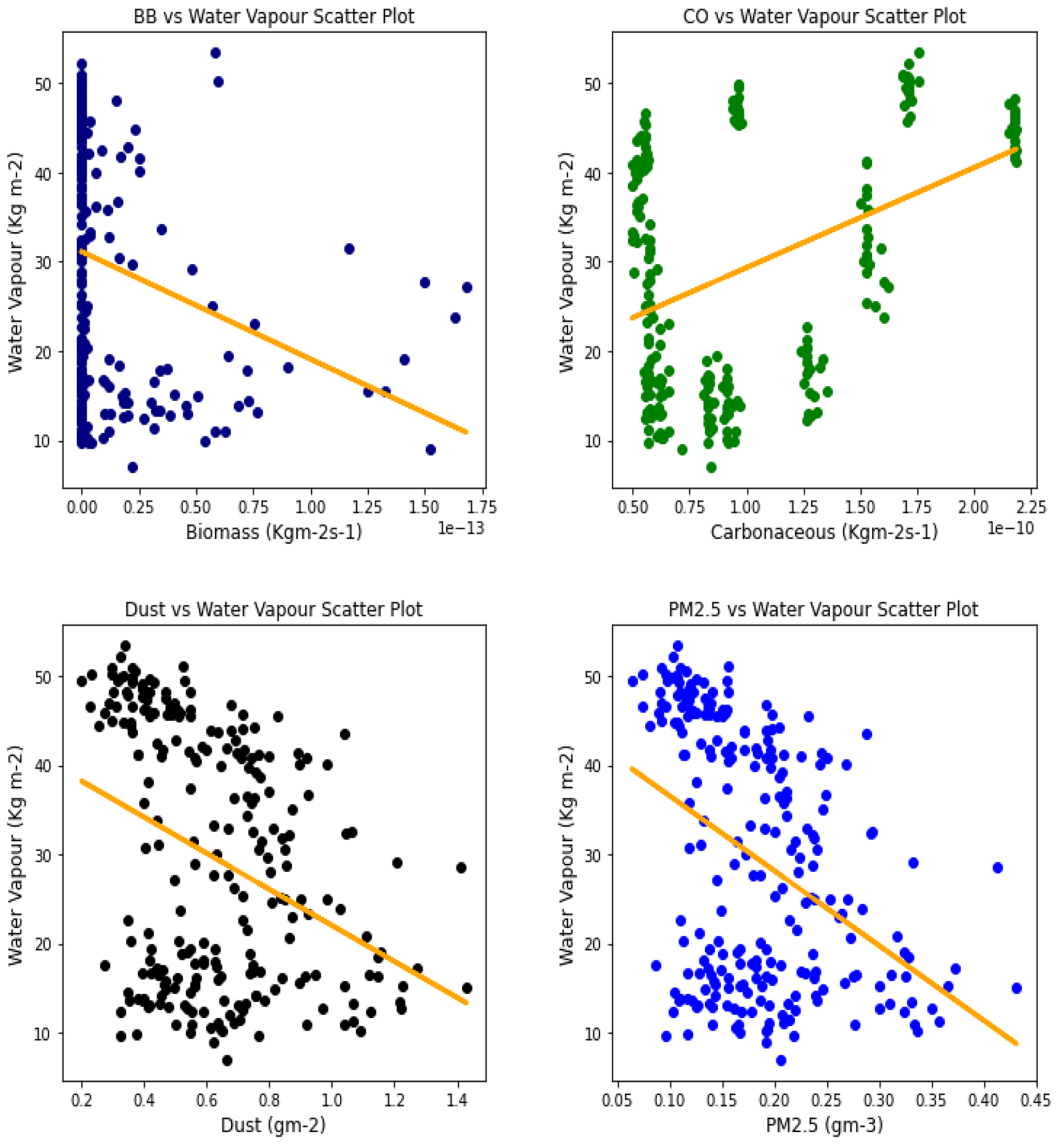
Correl. of aerosols and H_2_O vapor over Banizoumbou

**Fig. 22:**
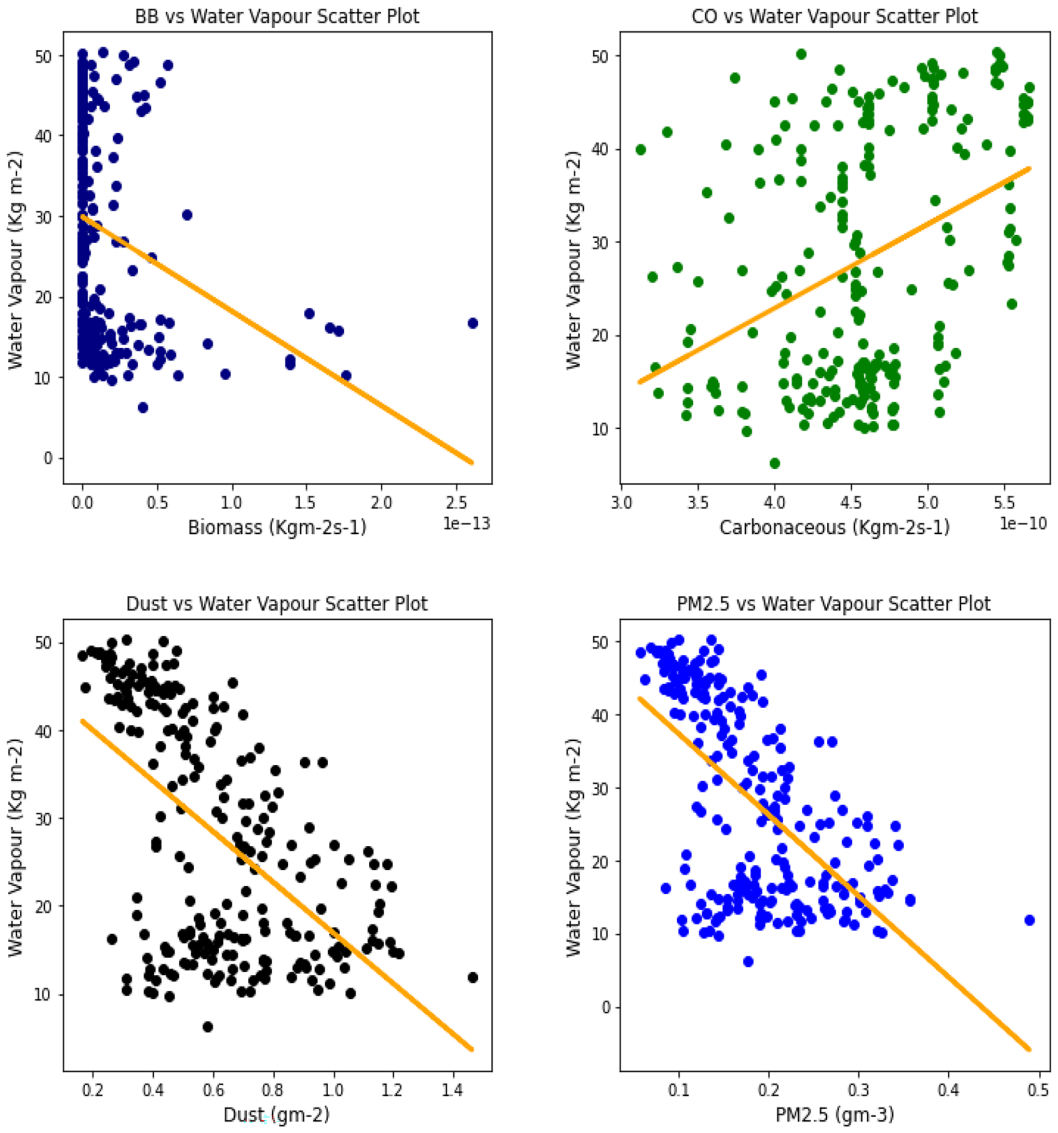
Correl. of aerosols and H_2_O vapor over Kano

**Fig. 23:**
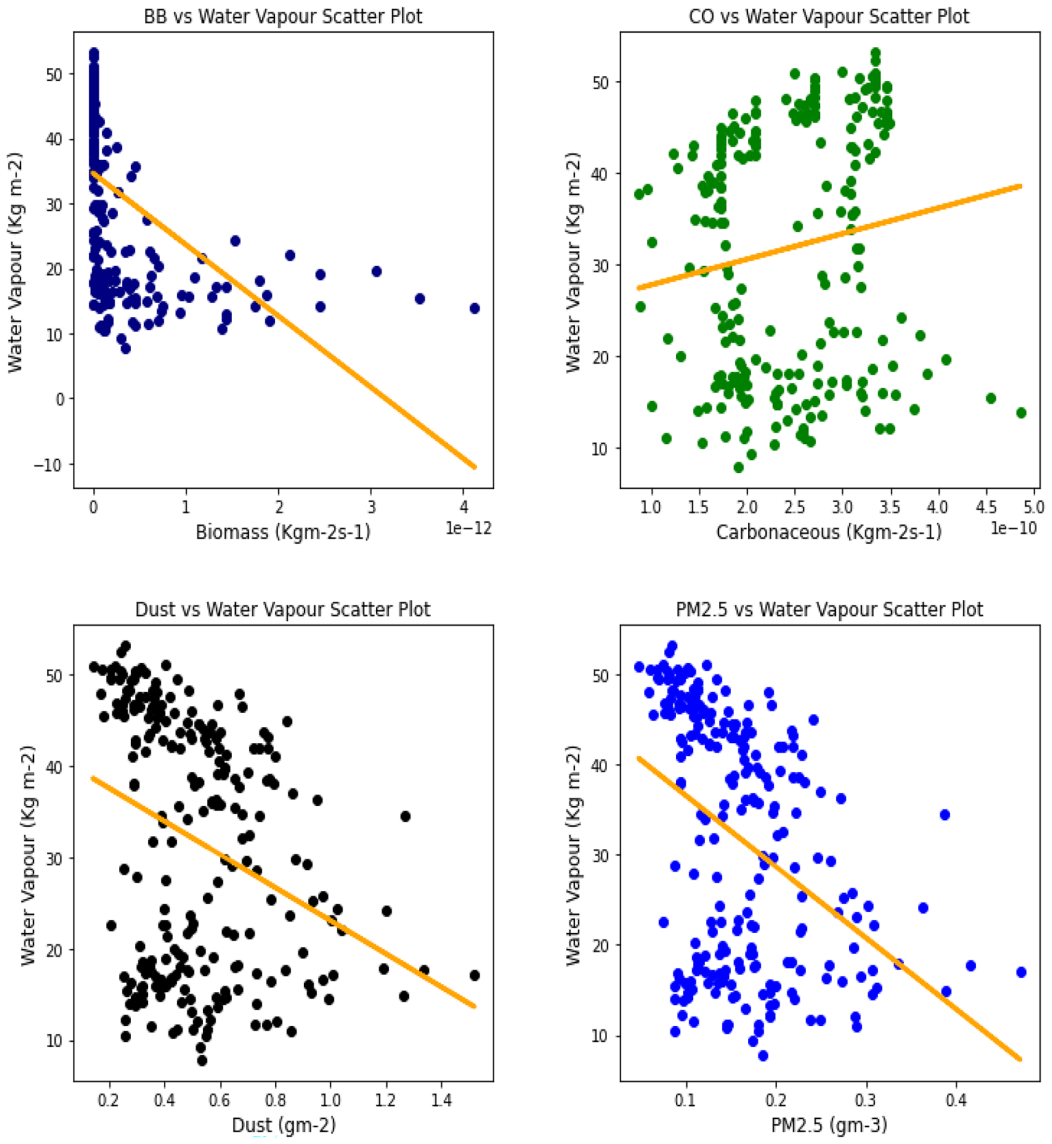
Correl. of aerosols and H_2_O vapor over Ouagadougou

The correlation values between aerosol types and wind speed (Table 1) showed less relationship; it can be assumed that the higher the wind, the less the presence of these aerosol types. The aerosol can be said to have been transported from such a location, thus giving a low relationship. This is in support of Danjuma et al.’s (2021) study which showed the transportation of aerosol from central Africa to West Africa over maritime. Dust and PM_2.5_ showed a strong relationship with wind speed over Kano with correlation values of 0.63 and 0.69, respectively. For carbonaceous, Banizoumbou and Kano recorded a strong correlation between the aerosol type and wind speed with values of − 0.62 and − 0.51. The same cannot be said for biomass burning which showed no significant relationship with wind speed over all the locations for this study. The *p* value also corroborated the statistical result by showing that under biomass burning, there was no relationship between it and the wind speed in Banizoumbou and Freetown. This is evidenced in its *p* value which is significantly greater than the significant value of 0.05. Hence, the null hypothesis (*H*_o_) is accepted. For carbonaceous, the null hypothesis was accepted in Freetown and Ikeja, thus translating that there is no relationship between wind speed and carbonaceous. Dust and PM_2.5_ exhibited some level of relationship between them and the wind speed. The dust results are in support of studies by Knippertz (2008) and Schepanski et al. (2009) in which strong wind from line squalls can trigger dust mobilization.

**Table 1:** Summary of the result of scatter plots of aerosol types and wind speed

Stations	Biomass burning		Carbonaceous		Dust		Particulate matter 2.5	
	*r*-value	*p* value	*r* value	*p* value	*r* value	*p* value	*r* value	*p* value
Accra	−0.30	1.72e−06	−0.34	7.55e−08	−0.32	2.87e−07	−0.38	1.74e−09
Banizoumbou	0.05	0.47	−0.62	1.04e−26	0.38	1.05e−09	0.43	5.17e−12
Freetown	−0.03	0.60	−0.04	0.56	−0.34	4.44e−08	−0.43	5.68e−12
Ikeja	−0.23	0.00	−0.04	0.55	−0.38	7.22e−10	−0.46	3.81e−14
Kano	0.22	0.00	−0.51	3.98e−17	0.63	2.05e−28	0.69	4.74e−35
Ouagadougou	0.29	5.21e−06	−0.42	1.15e−11	0.38	1.11e−09	0.44	1.17e−12

For the relationships between aerosol types and convective precipitation in Table 2, it showed that all locations recorded a negative correlation in biomass, dust, and PM_2.5._ This shows that the presence of precipitations leads to a decrease in the amount of aerosol types present in the atmosphere, and vice-versa. This is in support of Wu et al.’s (2011) study which established that investigation of regional modeling has shown that the radiative effect could cause an overall reduction in precipitation but may increase nighttime precipitation and also intensify extremely high precipitation rates (Kolusu et al., 2015). The strongest relationships between convective precipitations and aerosol types can be observed in Accra (for Carbonaceous) at 0.60, Kano (for dust) at −0.66, and Freetown (for PM_2.5_) at −0.71. Biomass burning did not record any strong relationship with convective precipitation in any of the locations, with the highest value being −0.32 at Ouagadougou. Relating the probability value to the significance test of 0.05, it can be deduced that all aerosol types have a probability value less than the significant value. This means that the null hypothesis is rejected, thus stating that a relationship exists between convective precipitation and aerosol types except over Accra where convective precipitations and PM_2.5_ recorded a probability value of 0.84, which makes it greater than the significant value, thus making the null hypothesis to be accepted, which translates to saying that there exists no relationship between the two variables over Accra. This anomaly in Accra as compared to other locations, especially regions within the same climatic borders, cannot be said to be unrelated to the anomaly in weather situations as detailed by Acheampong (1982).

**Table 2:** Summary of the result of scatter plots of aerosol types and convective precipitation

Stations	Biomass burning		Carbonaceous		Dust		Particulate matter 2.5	
	*r*-value	*p* value	*r*-value	*p* value	*r*-value	*p* value	*r*-value	*p* value
Accra	−0.13	0.01	−0.03	2.11e−07	0.11	0.03	−0.01	0.84
Banizoumbou	−0.25	3.23e−06	0.60	9.24e−37	−0.55	6.81e−30	−0.52	3.62e−26
Freetown	−0.18	0.00	−0.17	0.00	−0.63	4.24e−41	−0.71	7.59e−56
Ikeja	−0.24	3.46e−06	0.33	6.27e−11	−0.19	0.00	−0.34	5.13e−11
Kano	−0.15	0.00	0.19	0.00	−0.66	8.18e−47	−0.62	9.12e−40
Ouagadougou	−0.32	6.92e−10	0.40	6.17e−15	−0.53	4.85e−28	−0.53	8.15e−28

The relationship between the aerosol types and water vapor as seen in Table 3 showed that PM_2.5_ had strong relationships with water vapor in 4 locations, with Banzioumbou and Ouagadougou recording weak correlations at −0.41 and −0.40, respectively. All the rainforest-categorized stations of Accra, Ikeja, and Freetown recorded correlation values of −0.69, − 0.66, and −0.71, respectively. Kano was the only station with a strong correlation value of −0.61 among the Savannah stations. Dust occurrence and water vapor correlation showed Kano, Ikeja, and Accra recording strong and negative relationships. There was no strong relationship value recorded for any station under carbonaceous, while only Accra had a strong correlation between water vapor and biomass burning with a value of −0.56. It is important to state that all correlations between water vapor and 3 aerosol types (biomass burning, dust, and PM_2.5_) were negative correlations, but only Accra and Freetown recorded a negative correlation with Carbonaceous. The *p* value showed that there exists a relationship between all the aerosol types and water vapor except in Freetown (for biomass burning and carbonaceous) and Ikeja (for carbonaceous only). This outcome suggests that all Savannah locations showed signs of an existing relationship between water vapor and all pollutants.

**Table 3:** Summary of the result of scatter plots of aerosol types and water vapor

Stations	Biomass burning		Carbonaceous		Dust		Particulate matter 2.5	
	*r*-value	*p* value	*r*-value	*p* value	*r*-value	*p* value	*r*-value	*p* value
Accra	−0.56	1.56e−21	−0.48	1.59e−15	−0.53	4.94e−19	−0.69	1.03e−34
Banizoumbou	−0.29	5.23e−05	0.41	2.20e−11	−0.35	2.28e−08	−0.41	4.12e−11
Freetown	−0.05	0.45	−0.05	0.43	−0.48	2.25e−15	−0.66	1.97e−31
Ikeja	−0.44	1.32e−12	0.00	0.99	−0.60	4.84e−25	−0.74	7.39e−43
Kano	−0.28	7.90e−06	0.39	6.45e−10	−0.56	7.48e−21	−0.61	2.36e−25
Ouagadougou	−0.49	1.76e − 14	0.15	0.02	−0.32	6.29e−07	− 0.40	9.63e−11

## Conclusion

The pronounced presence of pollutants in the atmosphere during the dry months is a confirmation of a real-life scenario in which low visibility in the atmosphere is a result of these pollutants since the visibility reduction in the wet months can be a result of convective activities. Dust presence all through the year at the point source (Bodélé depression) and possible spreading down south under favorable north-easterly wind can lead to respiratory-related problems with its continued presence in the atmosphere. The results also present the same spatial pattern between biomass/carbonaceous and dust/PM_2.5_. This shows that dust and PM_2.5_ may not necessarily be separable by ordinary eyes while some studies have shown the possibility of both biomass and carbon happening simultaneously especially when biomass burning takes place. The daily heavy traffic situation and continuous emission of CO by industries in Ikeja may be responsible for the high carbon pronouncement over the location in the months of March/April/May (MAM), but biomass burning is not as evident as carbon does. Though biomass burning aerosol and carbon are said to exist together once one of them is present in the atmosphere, the result over Ikeja showed otherwise in which carbon was present and biomass burning was not. It means they can also exist separately. The constant traffic situation in Lagos as well as the high number of industries, which is one of the reasons the state is regarded as the commercial hub of West Africa, can be said to be the reason for the continuous presence of carbonaceous all through the months of the year over Ikeja. The correlation output showed that while most aerosol types exhibited a statistically proven relationship at a particularly significant level, CO with water vapor over Ikeja, PM_2.5_ with convective precipitation over Accra, and BB and CO with wind speed over Freetown and Ikeja did not have any statistical relationship. In summary, it can be concluded that most aerosols can either be positively or negatively correlated with meteorological variables, and this can have an effect on the atmospheric composition or formation.

## Data Availability

The datasets generated during and/or analyzed during the current study are available from the corresponding author upon reasonable request.
